# NAT10-mediated N4-acetylcytidine (ac4C) modification of PIK3R2 mRNA promotes malignant progression of glioblastoma

**DOI:** 10.1038/s41419-025-08328-y

**Published:** 2025-12-17

**Authors:** Xiannan Meng, Tao Dong, Jiayu Xu, Kaiwen Tian, Wendong Yang, Zixuan Liu, Yongjing Qian, Dingding Liu, Changxiu Chen, Jin Bai, Hongmei Yong, Xiaojin Wu, Zhigang Shen

**Affiliations:** 1 Cancer Institute, Cellular Therapeutics School of Medicine, Xuzhou Medical University, Xuzhou, Jiangsu China; 2 Department of Oncology, The Affiliated Huai’an Hospital of Xuzhou Medical University and The Second People’s Hospital of Huai’an, Huai’an, Jiangsu China; 3 Department of Neurosurgery, The Affiliated Hospital of Xuzhou Medical University, Xuzhou, Jiangsu China; 4 Department of Pediatrics, The Affiliated Huaihai Hospital of Xuzhou Medical University, Xuzhou, Jiangsu China; 5 Center of Clinical Oncology, The Affiliated Hospital of Xuzhou Medical University, Xuzhou, Jiangsu China; 6 Jiangsu Center for the Collaboration and Innovation of Cancer Biotherapy, Cancer Institute, Xuzhou Medical University, Xuzhou, Jiangsu China; 7 Cancer Center, Xuzhou Central Hospital, Southeast University, Xuzhou, Jiangsu China; 8 Department of Radiation Oncology, Xuzhou Clinical School of Xuzhou Medical University, Xuzhou, Jiangsu China

**Keywords:** Tumour biomarkers, Targeted therapies

## Abstract

Glioma is the most common primary tumor in the central nervous system, with glioblastoma (GBM) representing one of the most malignant forms, accounting for 46.1% of cases. GBM is characterized by rapid progression, high malignancy, and poor prognosis, with median survival remaining less than 15 months despite combined surgery, radiotherapy, and chemotherapy. NAT10, the only known acetyltransferase mediating N4-acetylcytidine (ac4C) modification in eukaryotes, has been implicated in promoting tumorigenesis and progression in colon cancer, bladder cancer, pancreatic ductal cancer, among others. In this study, we found that NAT10 is highly expressed in GBM and is positively correlated with malignant pathological features and poor prognosis in patients. In vitro experiments demonstrated that NAT10 promotes tumor proliferation, migration, and invasion. In vivo experiments further confirmed that NAT10 facilitates malignant progression of tumors. Mechanistically, we revealed that NAT10 regulates PIK3R2 stabilization through ac4C modification, thereby participating malignant characterization of GBM. Additionally, our data demonstrated a positive correlation between NAT10 and PIK3R2 in glioma patients. Taken together, our findings strongly suggest that NAT10 is a potential therapeutic target for GBM.

## Introduction

Glioma is the most common primary malignant tumor in the human central nervous system, with glioblastoma multiforme (GBM) being the most prevalent subtype, accounting for 46.1% of cases [1]. GBM is characterized by strong invasion, high malignancy, rapid progression, and poor prognosis. With advancements in molecular biology technology, molecular targeted therapy has emerged as a research hotspot and a new hope for the treatment of GBM patients [2]. Recent studies have indicated that RNA modification is a key regulatory mechanism involved in glioma genesis, progression, immune regulation, and therapeutic response. Understanding how RNA modification contributes to the malignant evolution of glioma has become a research focus, and targeting this process may provide a new therapeutic strategy for glioma [3].

mRNAs are known to transfer genetic information from DNA to proteins, undergoing multiple modifications during or after transcription [4]. Hence, reversible RNA modification is an important component of epigenetics, playing a crucial role in post-transcriptional regulation. RNA modification can affect a series of cellular processes, including RNA splicing, export, protein translation, and degradation [5].

N4-acetylcytidine (ac4C) modification is an ancient and conserved RNA chemical modification that aids in the accurate reading of specific nucleotide sequences during translation, stabilizes mRNA expression, and enhances transcription efficiency [6]. NAT10, the only known acetyltransferase to mediate ac4C modification, modifies tRNA with the assistance of the Tan1/THUMPD1 adapter to stabilize its structure [7]. Recent studies have linked ac4C modification to the occurrence and development of various diseases. For instance, RNA ac4C leads to cisplatin resistance in bladder cancer [8]; in multiple myeloma, ac4C stabilizes CEP170 mRNA expression to promote tumor proliferation [9]. Upregulation of MORC2 expression through protein acetylation enhances tumor resistance to chemoradiotherapy in breast cancer [10]. NAT10-mediated reduction of ac4C modification inhibits the stability and translational efficiency of BCL9L, SOX4, and AKT1 in bladder cancer, thus inhibiting tumor progression [11]. Additionally, NAT10 plays a role in promoting tumor progression in breast cancer [12], gastric cancer [13], and head and neck squamous cell carcinoma [14]. Recent research revealed that NAT10 promotes GBM malignancy by acetylating JARID2 mRNA, enhancing its stability and PRC2-mediated transcription, thereby driving tumor stemness and progression. [15]. While this recent publication provides important insights into the role of NAT10 in GBM, the molecular mechanisms by which NAT10 promotes GBM progression remain incompletely understood.

In epithelial tumors, epithelial-mesenchymal transition (EMT) is a key mechanism driving tumor cells to acquire migration and invasion abilities [16]. Previous reports have demonstrated that ac4C can promote EMT by regulating the abnormal transcription of EMT-related genes or modulating tumor microenvironmental factors. For example, NAT10 enhances COL5A expression through ac4C modification, promoting EMT in gastric cancer cells and leading to malignant progression [13]. Although GBM lacks classical epithelial features. it undergoes a functionally analogous process termed proneural–mesenchymal transition (PMT), which constitutes a central mechanism underlying its aggressiveness. PMT endows GBM cells with enhanced invasiveness, stem-like traits, therapeutic resistance, and the capacity to remodel the immunosuppressive microenvironment, collectively contributing to diffuse infiltration and recurrence. PIK3R2, a beta subunit of PI3K, is involved in the activation of the PI3K/AKT pathway and exhibits high expression in various tumors, including GBM [17]. It has been reported that lncRNA XLOC013218 promotes glioma cell proliferation and temozolomide (TMZ) resistance by targeting the PIK3R2-mediated PI3K/AKT pathway [18]. However, the regulatory mechanism of PIK3R2 overexpression in GBM remains unclear.

In this study, we report that NAT10 is highly expressed in glioma tissues, with high NAT10 expression positively correlated with increased tumor grade and poor prognosis in glioma patients. Additionally, knockdown of NAT10 inhibited the proliferation, migration, and invasion of GBM cells in vitro and suppressed malignant growth of GBM in vivo. Mechanistically, NAT10 upregulates ac4C modification of PIK3R2 by directly binding to its mRNA, enhancing its stability, thereby promoting malignant progression and invasiveness of GBM. Finally, we also confirmed a positive correlation between NAT10 and PIK3R2 expression in glioma patient tissues. This study provides new insights into the Nat10-mediated modulation of the malignant phenotype mechanism in GBM and offers theoretical support for NAT10 as a potential therapeutic target for GBM.

## Materials and methods

### Cell lines and cell cultures

The GBM cell lines U87, LN229 were cultured in DMEM medium (Gibco, USA) supplemented with 10% fetal bovine serum (FBS, Gibco, USA). All cell lines were obtained from the American Type Culture Collection (ATCC). Cells were incubated in a humidified atmosphere at 37 °C with 5% CO_2_.

### Patients and sample collection

The human tissue study was approved by the Ethics Committee of the Affiliated Hospital of Xuzhou Medical University, China, and was conducted in accordance with the Declaration of Helsinki. Tissue microarray (TMA) slides of 428 glioma tissues, 10 normal brain tissues, and 30 glioma tissues were registered in the Affiliated Hospital of Xuzhou Medical University from 2016 to 2024. The clinicopathological data of the patients were obtained from the medical records of the Affiliated Hospital of Xuzhou Medical University.

### Reverse transcriptase PCR (RT-PCR) and quantitative real-time PCR (qRT-PCR)

Total RNA was isolated from U87 and LN229 cell lines using Trizol (Yeasen, 19211ES60). The RNA was subsequently reverse-transcribed into cDNA and quantified via real-time PCR using ChamQ Universal SYBR qPCR Master Mix (Vazyme Biotech Co., Ltd, Nanjing, China). Relative expression levels of target genes were determined using the 2^−ΔΔCt^ method. All primers were synthesized by GENEWIZ Biological Technology (GENEWIZ, China), and their sequences are provided in Table S1.

### Cell viability CCK-8 assay

Cell proliferation was detected by CCK8 assay after cell counting according to the instructions of the CCK8 kit (Keygen Biotech, China).

### Plate clone formation assay

Glioma cells were extracted and digested into single-cell suspension with pancreatic enzymes, and cell counts were performed. Cells of appropriate density were inoculated into 6-well plates with 3 compound pores per subgroup. The plates were then cultured in a constant temperature incubator for 2–3 weeks, and the medium was changed every 2 days. When the clones were visible to the naked eye, the culture was terminated, and an appropriate amount of 4% paraformaldehyde was added for 20 min, followed by an appropriate amount of crystal violet dyeing solution for 15 min.

### Cell migration and invasion assays

The migration assay used Transwell chamber with a pore size of 8 µm using a 24-well plate (Corning, NY, USA) to assess cell migration ability. 1 × 10^4^ cells (U87) or 2 × 10^4^ cells (LN229) were inoculated with 200 µl DMEM in the upper chamber without FBS, and 800 µl medium containing 10% FBS was placed in the bottom chamber. The cells were incubated at 37 °C for 24 h and left at room temperature for 20 min with 4% paraformaldehyde (Biosharp, Hefei, China), then stained with 0.1% crystal violet (Beyotime, Shanghai, China) 20 min, cotton swab to wipe away excess cells in the upper layer. After being washed and dried by PBS, the cells were placed under a microscope (Olympus, Tokyo, Japan) and photographed in 5 randomly selected fields of view, followed by statistical analysis.

In the invasion test, serum-free medium dilution was used to prepare Matrigel (BD Bioscience, San Jose, CA, USA) at the ratio of 1:9, and 100 µL diluent was added to the upper layer of the cell and incubated at 37 °C for 1 h. The remaining experiment steps are the same as the transfer experiment.

### Plasmid transfection and stable transfection

All plasmids were purchased from GenePharma Technology (Shanghai, China). JetPRIME® Transfection Reagent (Polyplus, USA) was used for plasmid transfection according to the instructions of the product side. Stable cell lines were formed by lentivirus (Genechem) infection to form stable cells, and Puromycin was used for screening 6–9 h after infection.

### Actinomycin D assay

For the Actinomycin D assay, total RNAs extracted were treated with 1 µg/mL actinomycin D (Sigma-Aldrich, USA) to inhibit new RNA synthesis for 0, 1, 2, 3, and 4 h, respectively.

### Western blot

Total protein was isolated from the cells following various treatments. Briefly, cells were washed three times with PBS and lysed in an ice-cold extraction buffer (50 mM Tris-HCl pH 7.4, 150 mM NaCl, 1% NP, 0.1% SDS, and 1× protease inhibitor cocktail). Protein concentration was determined by the Bradford method. Proteins were separated using sodium dodecyl sulfate-polyacrylamide gel electrophoresis (SDS-PAGE) and electroblotted onto a polyvinylidene fluoride (PVDF) membrane (Roche, Basel, Switzerland) by standard procedures. Transferred blots were incubated sequentially with anti-NAT10 (Abcam, ab251186), anti-PIK3R2 (Proteintech, 67644-1-Ig), anti-FN1 (Proteintech, 15613-1-AP), anti-N-cadherin (Proteintech, 22018-1-AP), anti-Vimentin (Proteintech, 22031-1-AP), anti-Snail (Abcam, ab216347), anti-Tubulin (Proteintech, 11224-1-AP), anti-Phospho-Akt (Ser473) (Cell Signaling Technology, #4060), anti-AKT (Cell Signaling Technology, #9272) at 4 °C for 12 h, and HRP-conjugated secondary antibodies. Protein bands were visualized with an enhanced chemiluminescence detection kit and recorded on a radiographic film (Alpha Innotech, San Jose, CA, USA). Original western blots were provided in the Supplementary Material.

### Co-immunoprecipitation (Co-IP)

Cells were lysed using Western blot and immunoprecipitation (IP) lysis buffer (Beyotime, China) supplemented with protease inhibitor (MCE, USA). After centrifugation, the supernatant was collected and incubated with primary antibody at 4°C overnight. Subsequently, the lysate was incubated with 30 μL of protein A/G magnetic beads (MCE) at room temperature for 4 h. The collected beads-protein was washed six times and then subjected to analysis in subsequent experiments.

### RIP (RNA Immunoprecipitation) assays

The RIP assay was performed using the PureBinding^®^ RNA Immunoprecipitation Kit (GENESEED, P0101), following the manufacturer’s instructions. In brief, protein A/G magnetic beads were incubated with the primary antibody or IgG (MCE, USA) separately, and then incubated with cells. Subsequently, RNA was co-precipitated and extracted, and finally quantitated using qRT-PCR.

### Biotin RNA pull-down assay

Cells were lysed by ultrasonication in RIP buffer (150 mM KCl, 25 mM Tris-HCl, pH 7.4, 0.5 mM dithiothreitol, 0.5% NP-40) supplemented with protease inhibitors and RNase inhibitors. The cell lysates were then precleared with streptavidin magnetic beads (MCE, USA). In vitro transcribed biotin-labeled RNA or DNA probes adsorbed to streptavidin magnetic beads were subsequently incubated with cell lysates at 4 °C for 6 h before washing three times with RIP buffer. Finally, the potential interacting proteins were evaluated using Western blot analysis. The oligonucleotides for the RNA pull-down are listed in Supplementary Table S2.

### Animal experiments

Nude mice were placed in an air anesthesia machine cage and anesthetized with halothane. After skin disinfection, a 1 cm incision was made along the midline. The skull was fixed on the stereotactic frame 2 mm to the right of the anterior fontanel and 1 mm anterior to the coronal suture using a drilling device. Each nude mouse received an injection of 5 μl (8 × 10^6^ cells) into the right striatum. Following the injection, the incision was sutured, and the date of inoculation and cell type were noted on the cage. The growth of intracranial tumors was monitored at 0, 7, 14, and 21 days post-implantation. Fluorescein potassium was injected into the groin of the mice, and after a reaction time of 3–5 min, the mice were anesthetized and placed in an in vivo imager to observe the fluorescence value. When symptoms such as cachexia, hemiplegia, or mental confusion appeared, the mice were euthanized by systemic perfusion under anesthesia. The brains were then harvested and placed in neutral fixative for tissue preservation and subsequent analysis.

### IHC

Paraffin sections were initially deparaffinized and rehydrated. Antigen retrieval was performed, and endogenous peroxidase activities were blocked. Subsequently, the sections were incubated overnight at 4°C with the specified antibodies. Following this, the sections were incubated with a horseradish peroxidase-conjugated secondary antibody and subjected to DAB (3,3’-diaminobenzidine) staining. Finally, counterstaining was performed using hematoxylin.

### Available clinical data analysis

The clinical data for NAT10 and PIK3R2 were obtained from the Clinical Proteomic Tumor Analysis Consortium (CPTAC, https://proteomics.cancer.gov/programs/cptac), The Cancer Genome Atlas (TCGA, https://portal.gdc.cancer.gov/), and the Chinese Glioma Genome Atlas (CGGA, http://www.cgga.org.cn/).

Expression data for NAT10 from both normal and tumor samples in the CCGA database were downloaded. Boxplots were generated using the ggplot2 package (version 3.4.4) to compare the expression levels of NAT10 between normal and tumor samples. A *t*-test was conducted to assess the difference in NAT10 expression levels, with a *p* value of less than 0.05 considered statistically significant. Kaplan-Meier survival analysis was performed using the survival package (version 4.2.1) to evaluate survival differences between patients with high and low NAT10 expression. Survival curves were visualized using the survminer package, and a *p* value of less than 0.05 was considered statistically significant. The survival prediction ability of NAT10 at 1, 3, and 5 years was assessed using the timeROC package (version 0.4), and time-dependent ROC curves were plotted and visualized with the ggplot2 package.

Additionally, STAR-counts data and corresponding clinical information for glioma were downloaded from the TCGA database. The data were extracted in TPM format, log2-transformed (log2(TPM + 1)), and normalized. Samples with both RNA-seq data and clinical information were retained for analysis. The log-rank test was used to compare survival differences between the high and low NAT10 expression groups in the Kaplan-Meier survival analysis. Kaplan-Meier curves, *p* values, and hazard ratios (HRs) with 95% confidence intervals (CIs) were derived using the log-rank test and univariate Cox regression. All statistical analyses were performed using R software (version 4.0.3). A *p* value of less than 0.05 was considered statistically significant.

### Statistical analysis

GraphPad Prism Software (GraphPad Software, La Jolla, CA, USA) was used to perform statistical analysis. Two-tailed Student’s *t* test and one-way ANOVA analysis were performed for statistical comparisons. All statistics analysis data were expressed as mean ± standard error of the mean. A *p* < 0.05 was considered statistically significant.

## Results

### NAT10 is highly expressed in gliomas and positively correlated with malignant degree and poor prognosis

**Fig. 1:**
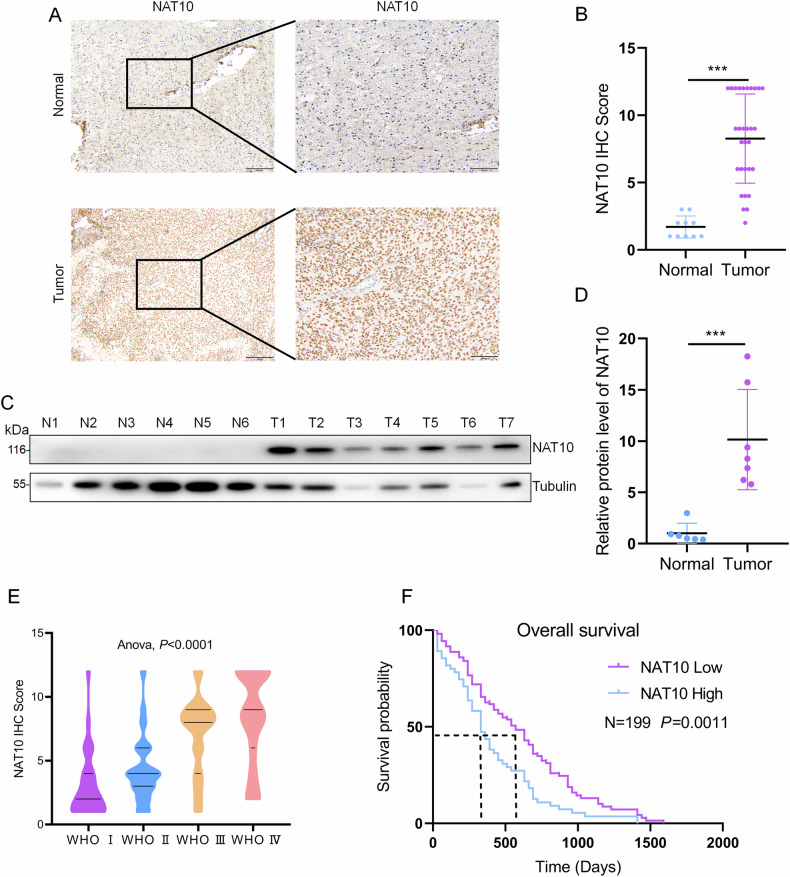
NAT10 is highly expressed in gliomas and positively correlated with poor prognosis. **A** Immunohistochemical staining of NAT10 in normal brain tissues (*n* = 10) and glioma tissues (*n* = 30). Representative images are shown. **B** Semi-quantitative scoring statistics of immunohistochemical staining for NAT10. **C** Western blot analysis of NAT10 expression in normal brain tissues (*n* = 6) and glioma tissues (*n* = 7). **D** Statistical results of gray scale analysis of NAT10 protein level. **E** Immunohistochemical staining of a glioma tissue microarray comprising 428 glioma samples, scored semi-quantitatively. **F** Kaplan-Meier survival analysis showing the relationship between NAT10 expression and overall survival (OS) in glioma patients. Patients with high NAT10 expression had significantly poorer OS compared to those with low NAT10 expression. ***p < 0.001.

To explore the role of NAT10 in the malignant progression of glioma, we first examined NAT10 expression in the CPTAC, CGGA, TCGA and REMBRANDT databases. The CPTAC database analysis revealed that NAT10 protein was significantly upregulated in GBM samples (Fig. S1A). Similarly, the CGGA database demonstrated that NAT10 mRNA expression was elevated in tumors, with levels increasing in accordance with tumor grade (Fig. S1B). Moreover, higher NAT10 expression was associated with poorer patient prognosis (Fig. S1C). The AUC curve analysis further indicated that NAT10 expression is a significant predictor of patient survival at 1, 3, and 5 years (Fig. S1D). TCGA database analysis showed that NAT10 was highly expressed in tumors (Fig. S1E), and glioma patients with high expression of NAT10 were classified as high-risk group. Survival curve analysis based on the risk model indicated that patients in the high-risk group had poor prognoses (Fig. S1F, G). The REMBRANDT database showed that NAT10 was also highly expressed in GBM patients (Fig. S1H). Immunohistochemistry (IHC) and Western blot analyses confirmed that NAT10 was highly expressed in glioma tissues compared to normal brain tissues (Fig. 1A–D), consistent with the database findings.

Next, we analyzed NAT10 expression in glioma patients using a TMA containing 428 glioma samples with clinical data (Fig. S1I). IHC results indicated that NAT10 expression was positively correlated with the WHO grade of glioma but not with gender, age, or pathological classification (Table 1). High NAT10 expression was significantly more frequent in high-grade gliomas (grades III and IV) than in low-grade gliomas (grades I and II) (Fig. 1E). Furthermore, Kaplan-Meier survival analysis was performed on 199 patients with available prognostic information. The results indicated that glioma patients with high NAT10 expression had shorter overall survival compared to those with low NAT10 expression (Fig. 1F). Collectively, these data demonstrate a correlation between high NAT10 expression and glioma malignancy and poor patient prognosis.

**Table 1:** NAT10 staining and clinicopathological characteristics of 428 patients.

Variables	Total	NAT10		χ2 value	*p* value
		Low (%)	High (%)		
All cases	428	210 (49.0)	218 (51.0)		
Gender					
Male	208	108 (51.9)	100 (48.1)	1.535	0.215
Female	220	102 (46.3)	118 (53.7)		
Age					
≤42	204	106 (49.5)	98 (17.4)	1.308	0.253
>42	224	104 (46.4)	120 (53.5)		
WHO grade					
Benign (Ⅰ-Ⅱ)	221	145 (65.6)	76 (34.4)	59.714	**<0.001**
Malignant (Ⅲ-Ⅳ)	207	65 (31.4)	142 (68.6)		
Histological type					
Glioblastoma	25	15 (60.0)	10 (40.0)	7.879	0.163
Astrocytoma	90	49 (54.4)	41 (45.6)		
Oligodendroglioma	15	6 (40)	9 (60)		
Ependymoma	4	4 (100)	0 (0)		
Medulloblastoma	14	6 (42.9)	8 (57.1)		
Gliocytoma	280	130 (46.4)	150 (53.6)		

The data shown in bold are statistically significant.

### Knockdown of NAT10 inhibits the proliferation of GBM cells in vitro

**Fig. 2:**
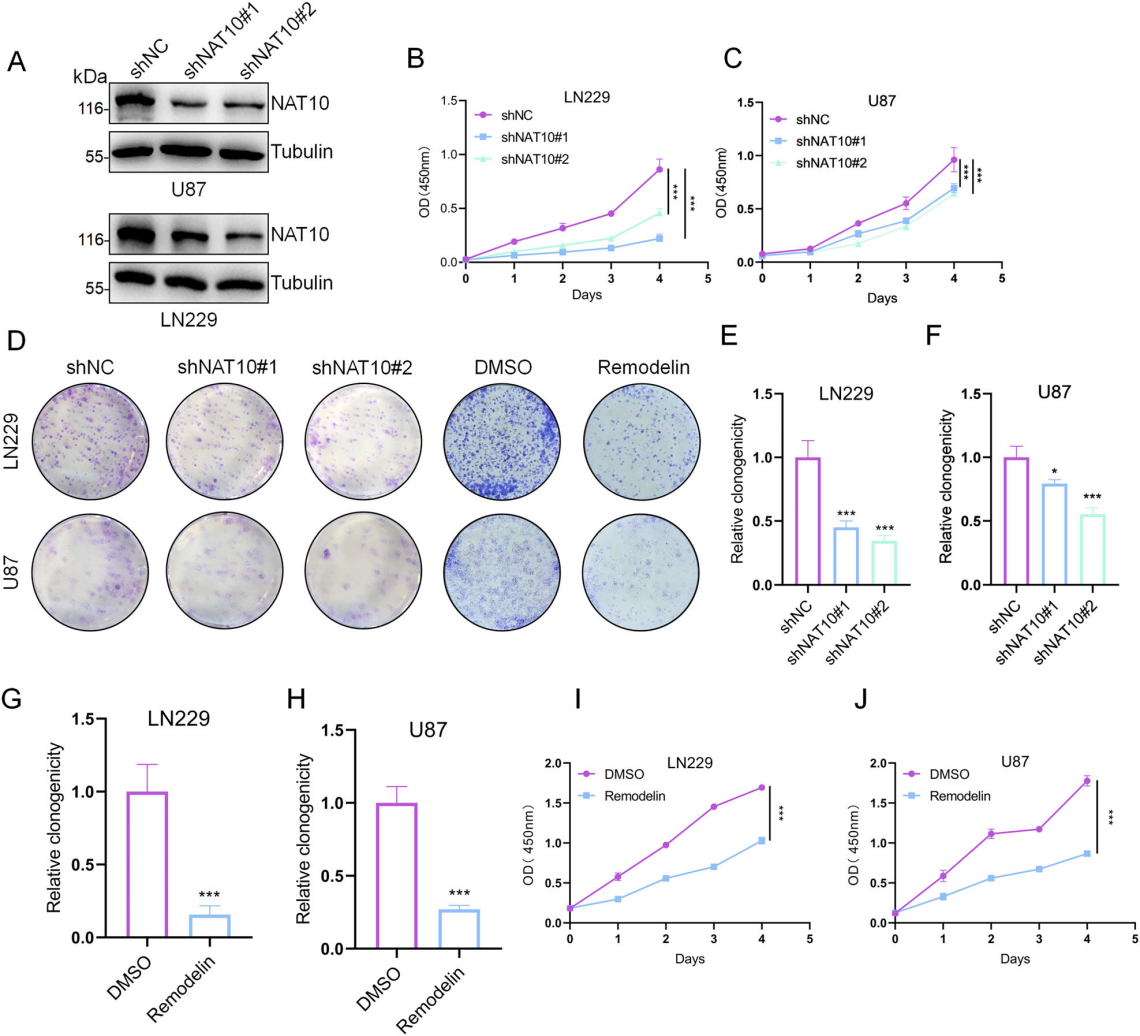
Knockdown of NAT10 inhibits the proliferation of GBM cells in vitro. **A** Western blot analysis showing the efficiency of NAT10 knockdown in LN229 and U87 cells. **B**, **C** CCK-8 assays were performed on U87 and LN229 cells with NAT10 knockdown for 4 days. **D**–**H** Colony formation assays were conducted to evaluate the proliferation ability of U87 and LN229 cells following NAT10 knockdown or treatment with Remodelin (20 μM for 24 h). **I**, **J** CCK-8 assays were performed on U87 and LN229 cells with Remodelin treatment (20 μM for 24 h). Error bars represent the mean ± SD. *n* = 3. ***p < 0.001, *p < 0.05. (Student’s *t* test).

Given that NAT10 is highly expressed in glioma patients, we investigated whether NAT10 affects the proliferation of GBM cells. NAT10 shRNA was used to knock down NAT10 expression in LN229 and U87 cells, and Western blot assays confirmed the knockdown efficiency (Fig. 2A). CCK-8 assays showed that NAT10 knockdown inhibited the proliferation of LN229 and U87 cells (Fig. 2B, C). Similarly, colony formation assays indicated that NAT10 knockdown reduced the colony formation ability of these cells (Fig. 2D–F).

Remodelin, a small molecule that specifically targets and inhibits NAT10, was used to treat LN229 and U87 cells. Colony formation and CCK-8 assays demonstrated that Remodelin inhibited the proliferation of LN229 and U87 cells (Fig. 2G–J). These results suggest that NAT10 promotes GBM cell proliferation in vitro.

### Knockdown of NAT10 inhibits migration and invasion of GBM cells in vitro

**Fig. 3:**
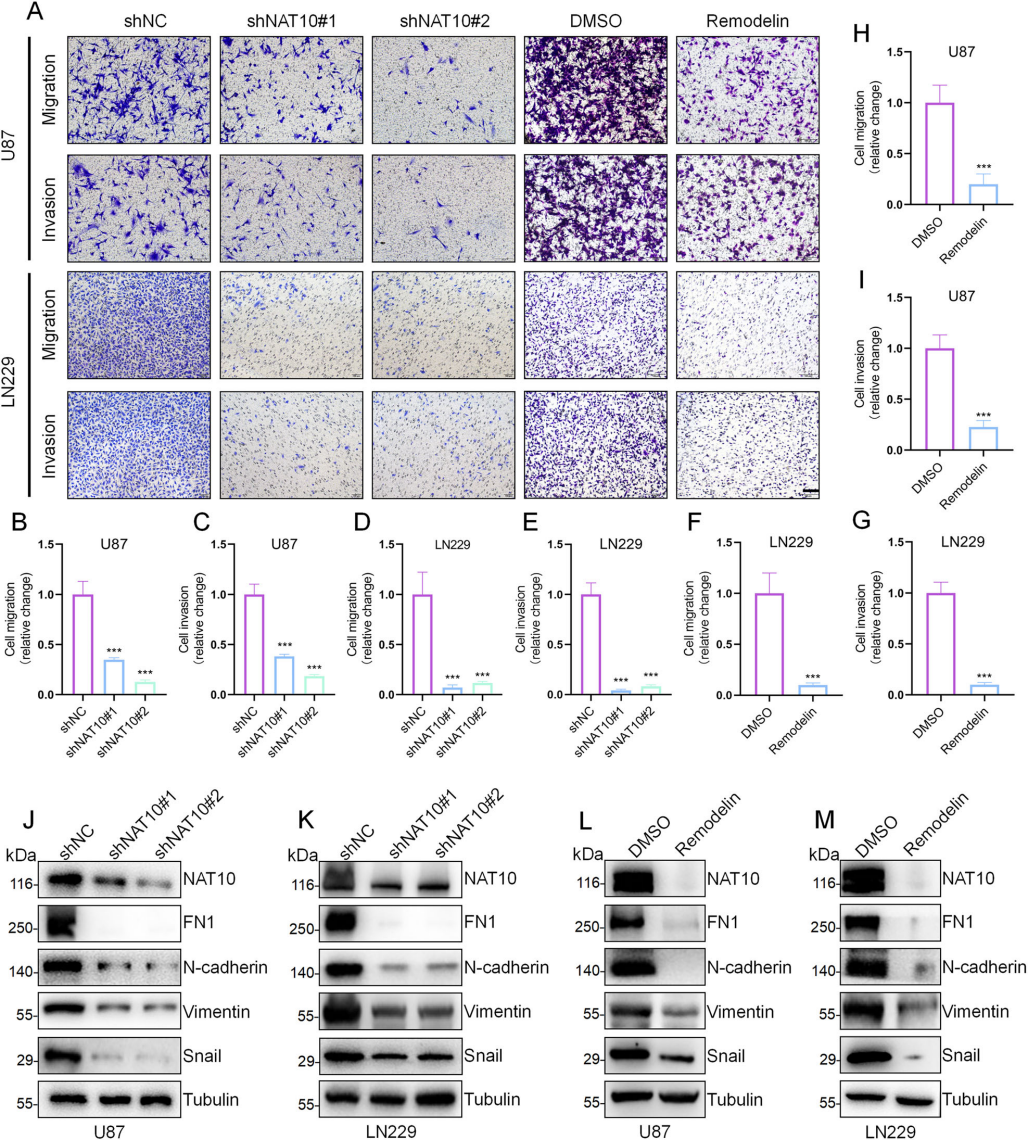
Knockdown of NAT10 inhibits migration and invasion of GBM cells in vitro. **A**–**I** Transwell assays were performed to assess the migration and invasion abilities of GBM cells following NAT10 knockdown or treatment with Remodelin (20 μM for 24 h). **J**, **K** Western blot analysis of FN1, N-cadherin, Vimentin, and Snail expression in U87 and LN229 cells after NAT10 knockdown. **L**, **M** Western blot analysis of FN1, N-cadherin, Vimentin, and Snail expression in GBM cells treated with Remodelin (20 μM for 24 h). Error bars represent the mean ± SD. *n* = 3. ***p < 0.001. (Student’s *t* test).

In gliomas, PMT is crucial for the diffuse and invasive phenotypes and is strongly associated with glioma recurrence and malignant progression [19]. Given that hypersensitive regulators of PMT could be potential targets for diffuse infiltrating glioma [20], we evaluated the role of NAT10 in regulating GBM cell migration and invasion. Transwell assays showed that NAT10 knockdown reduced the migration and invasion abilities of LN229 and U87 cells (Fig. 3A–E). Remodelin treatment also inhibited LN229 and U87 cell migration and invasion (Fig. 3A, F–I).

We then examined classical mesenchymal markers markers [21–23] and found that NAT10 knockdown in LN229 and U87 cells significantly downregulated the protein expression of FN1, N-cadherin, Vimentin, and Snail (Fig. 3J, K). Similar results were obtained with Remodelin treatment (Fig. 3L, M). To address the low efficiency of shRNA knockdown and improve the gene silencing effect, we then used siRNA technology, which allowed us to more accurately assess the immediate effect of gene silencing on cells and compare it with the long-term effect of shRNA. The same results as shNAT10 were obtained after siNAT10 (Fig. S2A–G). These findings suggest that NAT10 regulates the invasiveness of GBM cells in vitro via modulating their mesenchymal phenotype.

### NAT10 enhances PIK3R2 mRNA stability through ac4C modification

**Fig. 4:**
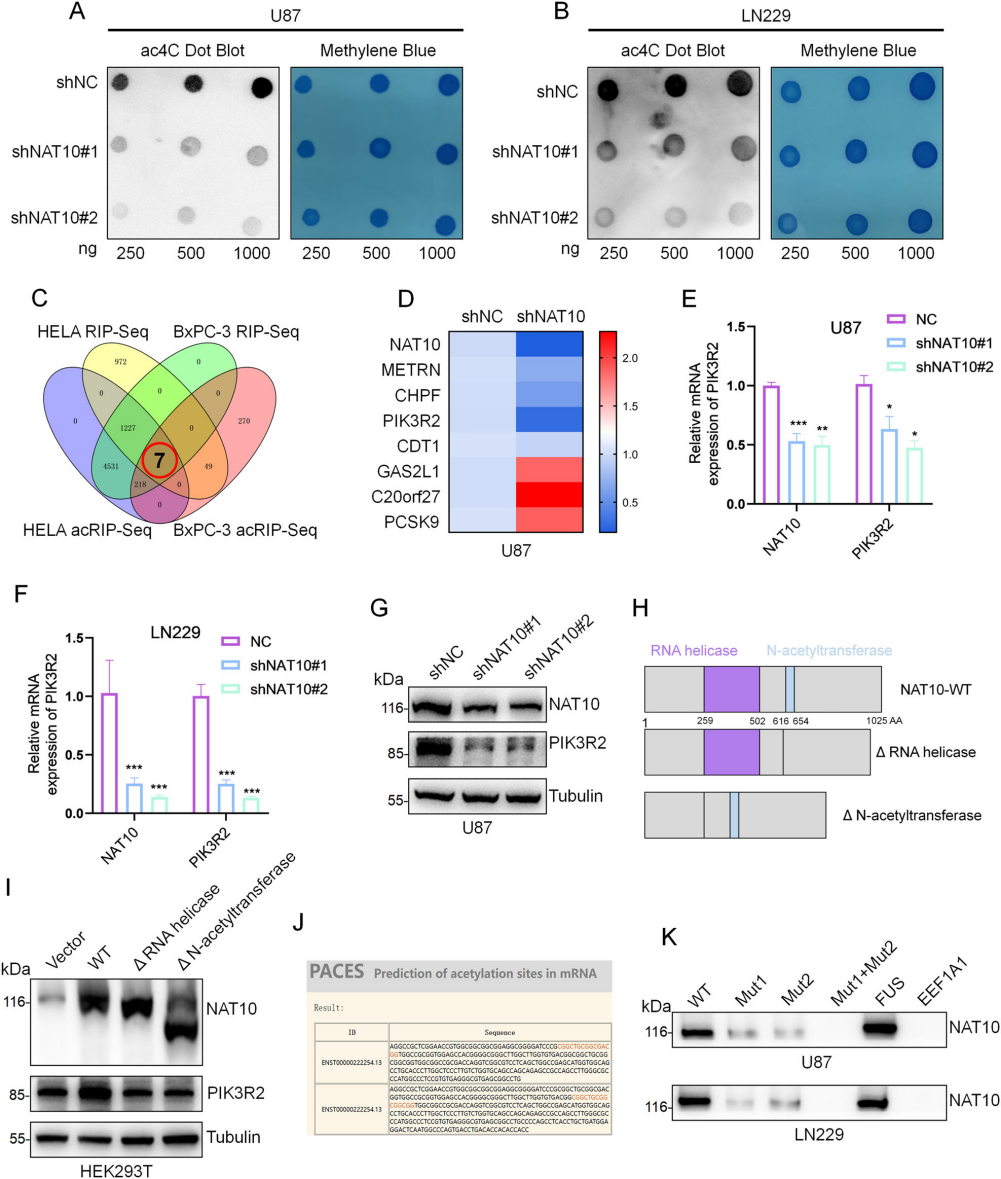
NAT10 positively regulates PIK3R2 expression in GBM cells. **A**, **B** Dot blot analysis of RNA ac4C levels following NAT10 knockdown in U87 and LN229 cells. **C** Retrieval and intersection of RIP-seq and acRIP-seq data from public databases for HeLa and BxPC-3 cells. **D** qPCR analysis of gene expression changes after NAT10 knockdown in U87 cells. **E**, **F** Changes in PIK3R2 expression levels assessed by qPCR following NAT10 knockdown. **G** Changes in PIK3R2 expression levels assessed by Western blot following NAT10 knockdown. **H** Schematic representation of the structure of the NAT10 protein, highlighting the RNA-binding region (purple box) and the N-acetyltransferase domain (blue box) within the truncated form of NAT10. **I** Western blotting was performed to detect the expression of PIK3R2 in HEK293T cells transfected with NAT10-WT, NAT10-ΔN-acetyltransferase, and NAT10-ΔRNA helicase. **J** The ac4C modification sites on PIK3R2 were predicted using the PACES website. **K** Mutant plasmids were constructed for RNA-Pull Down assays to verify specific binding sites, and FUS was selected as a positive reference and EEF1A1 as a negative reference. Error bars represent the mean ± SD. *n* = 3. ***p < 0.001, **p < 0.01, *p < 0.05. (Student’s *t* test).

NAT10 is known to regulate ac4C. To determine whether NAT10 promotes GBM cell malignant progression through this function, we performed dot blot hybridization experiments. Knockdown of NAT10 in U87 and LN229 cells decreased RNA ac4C modification, suggesting that NAT10 regulates RNA acetylation in GBM cells (Fig. 4A, B). To identify NAT10 ac4C target genes in GBM cells, we intersected RNA-seq and acRIP-seq data from HELA and BxPC-3 cells, identifying seven potential genes (Fig. 4C). qPCR assays showed that PIK3R2 was the most significantly downregulated gene following NAT10 knockdown, indicating that NAT10 may affect GBM progression by regulating PIK3R2 (Fig. 4D). Western blot and qPCR results confirmed that NAT10 positively regulates PIK3R2 expression in GBM cells (Fig. 4E–G and Fig. S3A–C). To further investigate the RNA acetylation-dependent function of NAT10, we constructed two modified forms of NAT10: a ΔN-acetyltransferase mutant, which lacks the catalytic N-acetyltransferase activity, and a ΔRNA helicase mutant, which has a truncated form that loses RNA-binding activity (Fig. 4H). In contrast to the wild-type NAT10, these two mutants failed to efficiently regulate PIK3R2 expression, suggesting that NAT10 modulates PIK3R2 expression through both its RNA-binding and acetyltransferase activities (Fig. 4I).

To explore how NAT10 regulates PIK3R2, we used the PACES website to predict acetylation sites in the PIK3R2 cDNA sequence and identified two highly conserved sites (Fig. 4J). RNA pull-down experiments confirmed that NAT10 directly binds to these predicted ac4C sites, but not to the mutated ones, suggesting that these sites are essential for the interaction between NAT10 and PIK3R2 mRNA (Fig. 4K and Fig. S4A). To further validate our findings in glioma, we conducted RNA pull-down experiments across multiple cancer types to assess the generalizability of ac4C regulation of PIK3R2 by NAT10. The results demonstrated a significant correlation between NAT10 and PIK3R2 expression in kidney cancer, colon cancer, and liver cancer. Additionally, the interaction between NAT10 and PIK3R2 was observed in both prostate and breast cancer, further supporting the broad applicability of this regulatory mechanism across different tumor types (Fig. S4B).

**Fig. 5:**
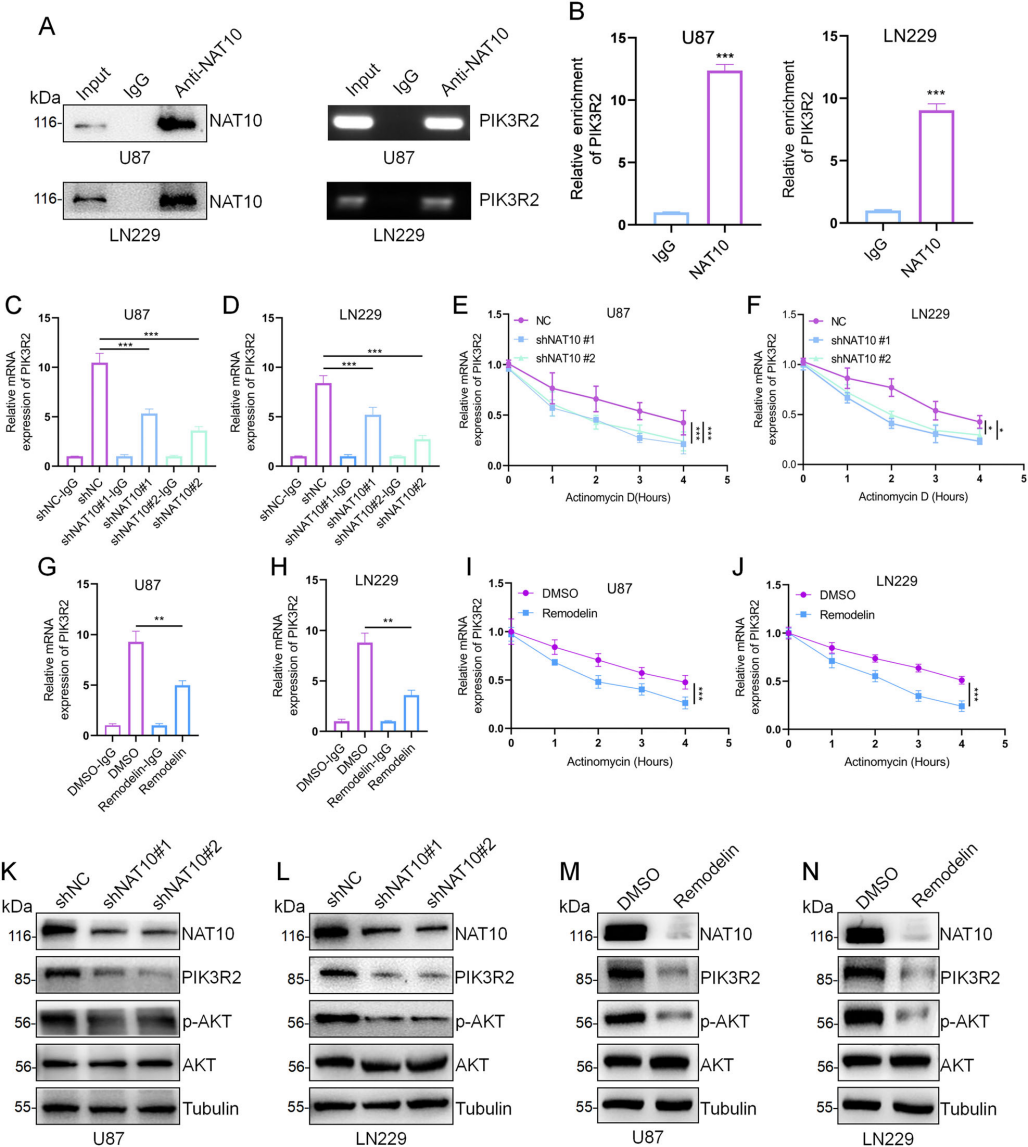
Regulation of PIK3R2 by NAT10 via ac4C. **A**, **B** RIP assays in U87 and LN229 cells using anti-NAT10 antibody. NAT10 protein was verified by Western blot, and the enrichment of PIK3R2 mRNA in NAT10 complexes was analyzed by gel electrophoresis and qPCR. **C**, **D** ac4C-RIP assay detecting mRNA levels of ac4C-modified PIK3R2 in GBM cells after NAT10 knockdown. **E**, **F** U87 and LN229 cells treated with actinomycin D (200 nM) post-NAT10 knockdown, with qPCR measurements of PIK3R2 mRNA stability at 0 h, 1 h, 2 h, 3 h, and 4 h. **G**, **H** ac4C-RIP assay detecting mRNA levels of ac4C-modified PIK3R2 in GBM cells after treated with Remodelin. **I**, **J** U87 and LN229 were tested for PIK3R2 stability by qPCR after Remodelin treatment and addition of actinomycin D. **K**, **L** Western blot analysis of p-AKT (Ser473), PIK3R2, and total AKT levels after NAT10 knockdown in U87 and LN229 cells. **M**, **N** Western blot analysis of p-AKT (Ser473), PIK3R2, and AKT levels following Remodelin (20 μM for 24 h) treatment in U87 and LN229 cells. Error bars represent the mean ± SD. *n* = 3. ***p < 0.001, **p < 0.01, *p < 0.05. (Student’s *t* test).

RIP and CO-IP experiments in U87 and LN229 cells showed interaction between NAT10 and PIK3R2 at the mRNA level (Fig. 5A, B and Fig. S4C, D). ac4C-RIP assays confirmed that NAT10 knockdown decreased ac4C-modified PIK3R2 mRNA levels (Fig. 5C, D). In addition, we also verified the results in other tumor cells and obtained the same result (Fig. S4E–I). qPCR results showed that downregulating NAT10 expression significantly suppressed PIK3R2 stability, indicating that NAT10 regulates PIK3R2 mRNA stability via ac4C modification (Fig. 5E, F). In addition, treatment of GBM cells with Remodelin produced similar effects, as the ac4C-modified PIK3R2 levels were reduced and its mRNA stability was also suppressed (Fig. 5G–J). The above results indicated that NAT10 may regulate the expression of PIK3R2 through ac4C modification.

### NAT10 affects the activation of the PI3K/AKT pathway by regulating PIK3R2

Given that the PI3K/AKT pathway is commonly activated in human tumor cells and involved in GBM pathogenesis [24], we investigated whether NAT10 could activate the PI3K/AKT pathway by regulating PIK3R2. Western blot analysis showed that AKT phosphorylation at Ser473, necessary for full AKT activation, decreased after NAT10 knockdown, whereas total AKT levels remained unchanged (Fig. 5K, L). Similar results were obtained with Remodelin treatment (Fig. 5M, N). These data suggest that NAT10 activates the PI3K/AKT pathway by regulating PIK3R2 in GBM.

### Upregulation of PIK3R2 expression is required for NAT10-regulated GBM cell function

**Fig. 6:**
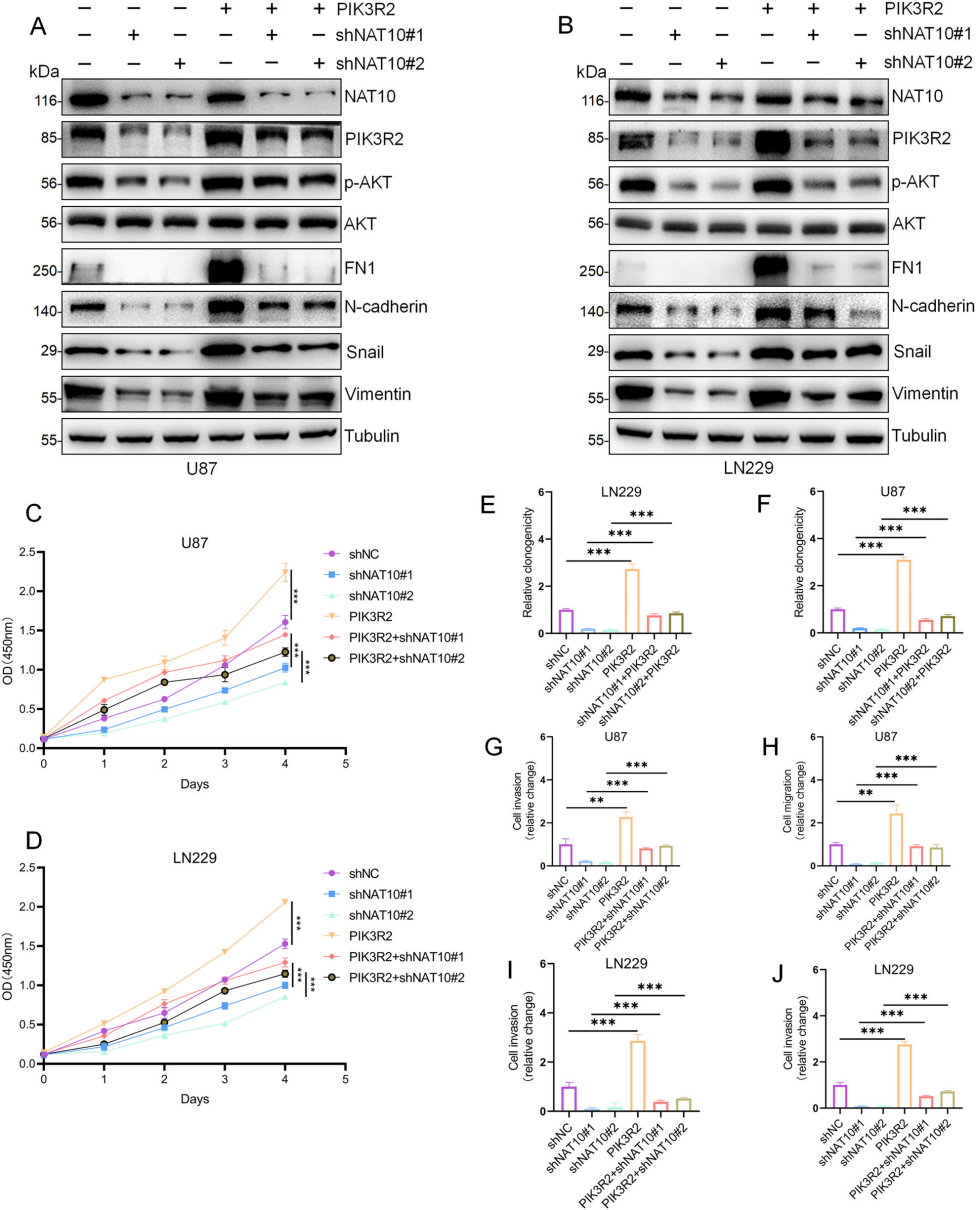
NAT10 participates in GBM malignant progression through PIK3R2. **A**, **B** Western blot analysis of protein expression levels of p-AKT (Ser473), AKT, FN1, N-cadherin, and Vimentin in U87 and LN229 cells with different treatments: shNC, shNAT10, shNAT10 + PIK3R2, and PIK3R2. **C**–**F** CCK-8 and colony formation assays assessing cell proliferation in U87 and LN229 cells under the same treatment conditions. **G**, **H** Transwell assays evaluating migration and invasion abilities of U87 and LN229 cells with various treatments: shNC, shNAT10, shNAT10 + PIK3R2, and PIK3R2. Error bars represent the mean ± SD. *n* = 3. ***p < 0.001. (Student’s *t* test).

We performed rescue experiments by overexpressing PIK3R2 in U87-shNAT10 and LN229-shNAT10 cells. Overexpression of PIK3R2 restored AKT phosphorylation and the expression of FN1, N-cadherin, Vimentin, and Snail, but did not affect total AKT levels (Fig. 6A, B). CCK-8 and colony formation assays showed that PIK3R2 overexpression restored tumor cell proliferation (Fig. 6C–F and Fig. S5B). Transwell assays indicated that PIK3R2 overexpression restored cell migration and invasion (Fig. 6G–J and Fig. S5A). These results suggest that NAT10 may promote the activation of the PI3K/AKT pathway via PIK3R2, thereby facilitating the associated mesenchymal phenotype changes and GBM malignant progression.

### NAT10 promotes GBM progression in vivo

**Fig. 7:**
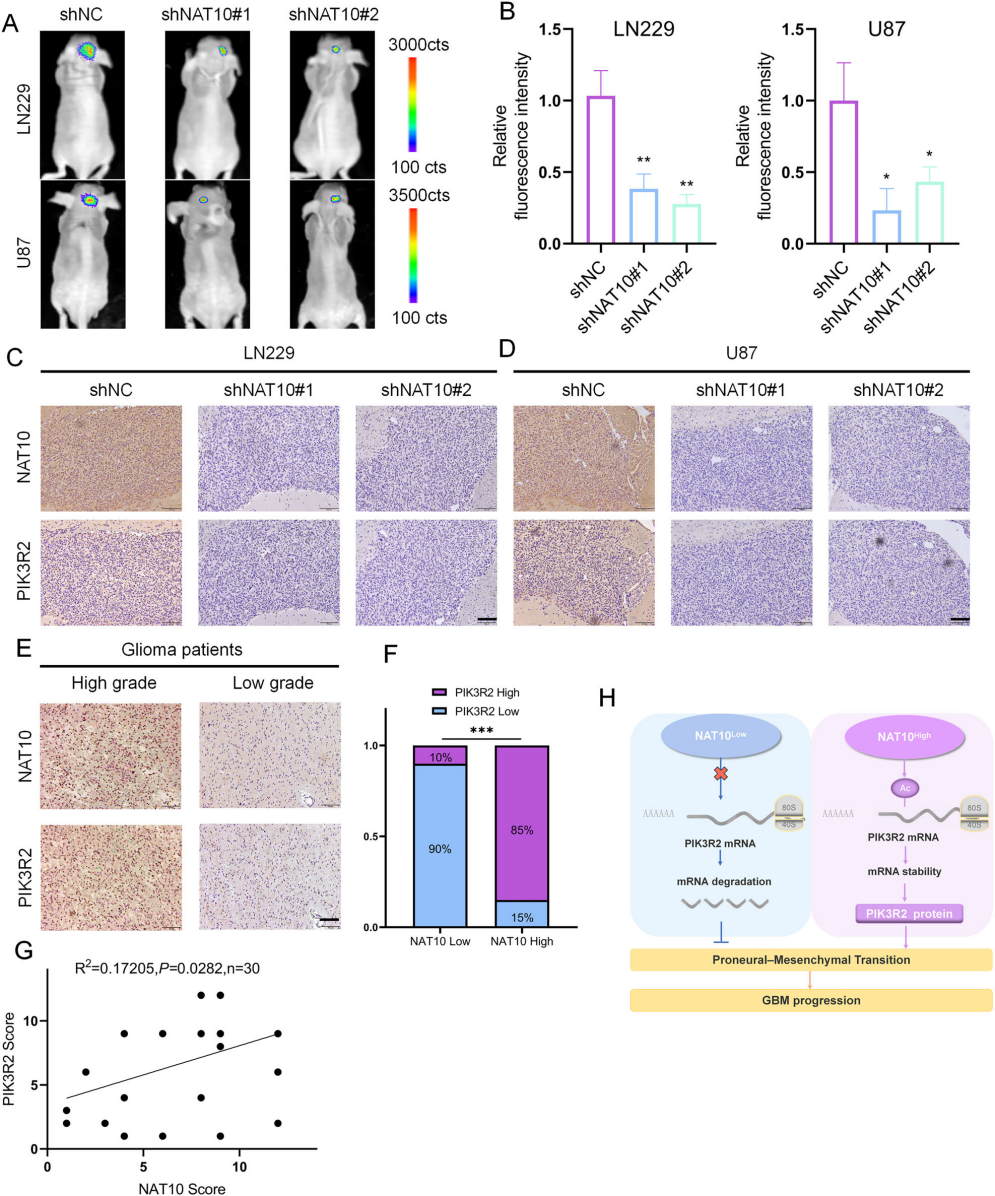
NAT10 promotes GBM malignant growth in vivo and correlates with clinicopathological features of glioma patients. **A**, **B** LN229 and U87 cells with shNC and shNAT10 were orthotopically transplanted into the right striatum of mice (*n* = 5 per group). Tumor growth was evaluated by measuring fluorescence intensity using an in vivo imaging system. Statistical analysis was performed using Student’s *t* test. **C**, **D** Immunohistochemical staining of NAT10 and PIK3R2 in orthotopic tumor tissues. **E** Representative images showing NAT10 and PIK3R2 expression in low-grade and high-grade tumor samples. **F** Semi-quantitative scoring of NAT10 and PIK3R2 staining (0–12 points) with chi-square test for correlation. **G** Pearson correlation coefficient analysis of NAT10 and PIK3R2 expression in 30 glioma samples based on immunohistochemical scores. **H** Mechanism diagram summarizing the findings: NAT10 directly regulates PIK3R2 via ac4C modification, leading to PMT and promoting GBM malignant development. **p < 0.01, *p < 0.05.

We investigated the effect of NAT10 on GBM tumor growth in vivo. U87-shNC, U87-shNAT10#1, and U87-shNAT10#2 cells were orthotopically injected into the brains of nude mice. Immunofluorescence intensity was monitored every 7 days. Mice were sacrificed upon development of cachexia, hemiplegia, confusion, and other neurological symptoms, and brain tissues were collected for IHC analysis. Fluorescence intensity was weaker in the U87-shNAT10 group than in the U87-shNC group. Similar results were observed in LN229-shNC and LN229-shNAT10 groups (Fig. 7A, B). IHC staining confirmed lower NAT10 and PIK3R2 expression in the U87-shNAT10 and LN229-shNAT10 groups (Fig. 7C, D).

Subsequently, we conducted in vivo recovery experiments and established LN229 and U87 cell lines with shNAT10, PIK3R2, and shNAT10 + PIK3R2 constructs. Compared to the NC group, the fluorescence intensity in the shNAT10 group was significantly reduced, while the PIK3R2 group exhibited the strongest fluorescence intensity. This observation aligns with previous reports indicating that PIK3R2 promotes tumor proliferation. The fluorescence intensity in the shNAT10 + PIK3R2 group was higher than that in the shNAT10 group (Fig. S6A–C). Regarding protein expression, PIK3R2 and p-AKT levels were lowest in the shNAT10 group, highest in the PIK3R2 group, and highest in the shNAT10 + PIK3R2 group. Notably, the expression of p-AKT in the shNAT10 + PIK3R2 group was higher than in the shNAT10 group (Fig. S6D, E). These in vivo data suggest that NAT10-mediated regulation of PIK3R2 is critical for GBM growth.

### Relationship between the NAT10-PIK3R2 axis and clinicopathological features of glioma patients

To further investigate the relationship between NAT10 and PIK3R2 expression and the clinical prognosis of glioma patients, we conducted a preliminary analysis using the TCGA database. The database showed that PIK3R2 was highly expressed in gliomas, and there was a positive correlation between NAT10 and PIK3R2 in gliomas (Fig. S7A, B). Finally, we explored the clinical implications of NAT10 and PIK3R2 functions in glioma. IHC analysis of 30 glioma tissue sections of different grades showed significantly higher NAT10 and PIK3R2 expression in high-grade gliomas than in low-grade gliomas (Fig. 7E). A positive correlation was found between NAT10 and PIK3R2 expression (Fig. 7F, G). These results indicate a clear association between the NAT10-PIK3R2 axis and the clinicopathological features of human gliomas. The final mechanism diagram summarizes our new findings: NAT10 regulates PIK3R2 stability through ac4C, leading to PMT and promoting GBM malignant development (Fig. 7H).

## Discussion

Accumulating evidence suggests that NAT10-mediated ac4C modification plays an important role in the development of human cancers [7]. Recent studies have demonstrated that NAT10 promotes GBM malignancy by enhancing the stemness of tumor cells through ac4C-mediated stabilization of JARID2 mRNA, thereby facilitating PRC2-dependent transcriptional regulation [15]. Consistent with these findings, our study further expands the understanding of NAT10’s oncogenic functions in GBM by identifying an alternative downstream target, PIK3R2. We show that NAT10 enhances the ac4C modification and stability of PIK3R2 mRNA, thereby contributing to the activation of the PI3K/AKT signaling pathway and promoting GBM proliferation and invasion. Together, these findings suggest that NAT10 exerts multifaceted regulatory roles in GBM progression by modulating the ac4C modification of multiple mRNA targets involved in tumor growth, stemness, and survival. Targeting NAT10 or its downstream ac4C-dependent pathways may thus represent a promising therapeutic strategy for GBM.

PMT has been increasingly recognized as a key contributor to tumor cells acquire enhanced invasiveness, stemness, and therapeutic resistance [25]. Our study highlighted the role of NAT10 in oncogenic functions such as proliferation and invasion of GBM cells. We observed that NAT10 was highly expressed in glioma tissues and its expression increased with the tumor grade. Additionally, high NAT10 expression was associated with poor prognosis in glioma patients. In vitro experiments demonstrated that the proliferation, migration, and invasion of GBM cells were inhibited following NAT10 knockdown, with a concurrent downregulation of PMT-related markers. The orthotopic xenograft model confirmed that NAT10 knockdown impeded the malignant growth of GBM cells in vivo.

NAT10 acts as an oncogene in various human cancers, being involved in cell proliferation, migration, and invasion [26]. Initially, many studies on NAT10 focused on its protein-acetyltransferase activity. For instance, NAT10 acetylates K767 of MORC2 to regulate the DNA damage-induced G2 checkpoint in breast cancer [10], and in colorectal cancer, it activates p53 at K120 by acetylation, counteracting the effects of MDM2 [27]. While recent reports indicate that NAT10 acetylates mRNA and that ac4C is the first acetylation event in mRNA catalyzed by NAT10 or its homologs, the role of ac4C in human diseases is significant [28]. For example, ac4C levels are higher in patients with cardiac hypertrophy [29] and are also implicated in renal ischemia-perfusion injury [30], interstitial cystitis [31], and cancer. Specifically, in cancer, ac4C inhibits ferroptosis by stabilizing FSP1 in colon cancer [32] and promotes malignancy and immunosuppression by stimulating FOXP1 to regulate tumor glycolysis metabolism in cervical cancer [33]. In this study, we found that mRNA acetylation was downregulated in GBM cells after NAT10 knockdown. We screened the potential downstream targets of NAT10 in GBM and confirmed that NAT10 directly binds to PIK3R2 for ac4C modification, affecting its stability.

PMT can be triggered by various cell signaling pathways, such as the NF-κB, STAT3, and TGF-β pathways [22, 34]. Among many signaling pathways, a large number of studies have clearly confirmed that the PI3K/AKT signaling pathway plays an indispensable role in driving the migration, invasion and cytoskeleton remodeling of GBM cells [35]. We investigated PIK3R2 expression in tumors and its potential relationship with clinical prognosis using TCGA data. While PIK3R2 was highly expressed in gliomas, we did not observe a significant correlation with patient prognosis. This may be due to factors such as the heterogeneity of prognostic data, tumor type/subtype differences, sample size limitations, and variations in data processing, all of which may obscure the relationship between PIK3R2 expression and prognosis. Nevertheless, PIK3R2 is a key signaling molecule likely involved in various tumor types and microenvironments. Previous studies have shown its role in glioma progression. Further studies with larger datasets and more detailed subgroup analyses are needed to better understand PIK3R2’s role in different tumors and its prognostic significance. Our study identified a novel mechanism where NAT10 mediates PIK3R2 to activate the PI3K/AKT pathway in GBM malignant proliferation and invasion. Knockdown of NAT10 decreased PIK3R2 and p-AKT expression, while overexpression of PIK3R2 post-NAT10 knockdown upregulated p-AKT. PMT-related marker proteins were also upregulated, restoring oncogenic functions. These results confirm that NAT10 promotes the malignant progression of GBM via PIK3R2.

Using siRNA to target oncogenes in vivo presents challenges. However, the small molecule Remodelin, with high selectivity and potency for NAT10, shows promise. Previous studies indicated an interaction between Remodelin and the acetyl-coa site on NAT10, antagonizing NAT10’s acetylation ability [36, 37]. Remodelin has been shown to reduce HIV copy numbers [38] and improve Hutchinson-Guilford premature senescence syndrome [39]. Additionally, Remodelin combined with cisplatin counteracts bladder cancer drug resistance [8] and inhibits EMT characteristics in hepatocellular carcinoma [40]. Our study observed that Remodelin inhibited GBM cell proliferation, migration, and invasion, and suppressed PIK3R2 and p-AKT expression in Remodelin-treated GBM cells, indicating that Remodelin regulates the PI3K/AKT pathway. These findings highlight Remodelin’s potential clinical application in glioma treatment.

## Conclusions

In summary, our study revealed that NAT10 promotes the malignant growth and aggressive activity of GBM and demonstrated that these functions depend on the activation of the PI3K/AKT pathway by directly binding PIK3R2 to ac4C modification, thus regulating its stability. This suggests that targeting NAT10 could be a potential therapeutic approach for glioblastoma patients.

## Supplementary information

Revised Supplementary Information

Orignal WB
