# Supplementary material for: NAT10-mediated N4-acetylcytidine (ac4C) modification of PIK3R2 mRNA promotes malignant progression of glioblastoma
Source: Cell Death Dis. 2025 Dec 17;17(1):106. doi: 10.1038/s41419-025-08328-y (PMC12848064; doi:10.1038/s41419-025-08328-y)
Supplement: Supplementary file 1 — Revised Supplementary Information [file 41419_2025_8328_MOESM1_ESM.docx]

NAT10-Mediated N4-Acetylcytidine (ac4C) Modification of PIK3R2 mRNA Promotes Malignant Progression of Glioblastoma

**Supplementary Methods**

The staining scores of NAT10 and PIK3R2 were blindly and independently performed by different pathologists. The signal was quantified based on the degree of positive cell staining and the percentage of staining. Staining intensity was scored from 0 to 3 (0= negative, 1= weak, 2= moderate, 3= strong), and the percentage of positively stained cells was also divided into four categories: 1 (0% to 25%), 2 (26% to 50%), 3 (51% to 75%), and 4 (76% to 100%). The IHC score levels of NAT10 and PIK3R2 staining were detected by IRS. The IRS score was calculated by comparing the staining intensity score with the percentage of positive cells. According to IRS, they were divided into low (IRS:0-6) and high (IRS:8-12).

**Supplementary Tables**

**Table1：NAT10 staining and clinicopathological characteristics of 428 patients.**

| Variables | Total | NAT10 | | χ2 value | *p* value |
| --- | --- | --- | --- | --- | --- |
|  |  | Low（%） | High（%） |  |  |
| All cases | 428 | 210(49.0） | 218(51.0) |  |  |
| Gender | | | | | |
| Male | 208 | 108 (51.9) | 100 (48.1) | 1.535 | 0.215 |
| Female | 220 | 102 (46.3) | 118 (53.7) |  |  |
| Age | | | | | |
| ≤42 | 214 | 106 (49.5) | 98 (17.4) | 1.308 | 0.253 |
| >42 | 224 | 104 (46.4) | 120 (53.5) |  |  |
| WHO grade | | | | | |
| Benign（Ⅰ-Ⅱ） | 221 | 145 (65.6) | 76 (34.4) | 59.714 | **＜0.001** |
| Malignant（Ⅲ-Ⅳ） | 207 | 65 (31.4) | 142 (68.6) |  |  |
| Histological type | | | | | |
| Glioblastoma | 25 | 15 (60.0) | 10 (40.0) | 7.879 | 0.163 |
| Astrocytoma | 90 | 49(54.4) | 41 (45.6) |  |  |
| Oligodendroglioma | 15 | 6(40) | 9 (60) |  |  |
| Ependymoma | 4 | 4(100) | 0 (0) |  |  |
| Medulloblastoma | 14 | 6 (42.9） | 8 (57.1) |  |  |
| Gliocytoma | 280 | 130 (46.4) | 150 (53.6) |  |  |

**Table S1: The primers used for qPCR in this study.**

| Name | Forward（5’-3’） | Reverse（3’-5’） |
| --- | --- | --- |
| NAT10 | GGATTGCCTCAACATCACTCGG | CGTTGGAGGAAAACTTCAGAGGC |
| PIK3R2 | ATGGCACCTTCCTAGTCCGAGA | CTCTGAGAAGCCATAGTGCCCA |
| GAPDH | CAAGGTCATCCATGACAACTTTG | GTCCACCACCCTGTTGCTGTAG |
| METRN | GCCCACGGTCTCGGCGTAGA | CACCACAGTGATGACAGACTCC |
| CHPF | AACGCACGTACCAGGAGATCCA | GGATGGTGCTGGAATACCCACG |
| CDT1 | GGAGGTCAGATTACCAGCTCAC | TTGACGTGCTCCACCAGCTTCT |
| GAS2L1 | TGCCCTGACCAGTTTCCCATGA | GTCGTGCTTGTCCAGGTAATGC |
| C20orf27 | GTCCTCAAAGAGGAGATACTGCT | GTGCTCTGAGTCGTATTCCAGC |
| PCSK9 | GACACCAGCATACAGAGTGACC | GTGCCATGACTGTCACACTTGC |

**Table S2: The sequences used in RNA pull down.**

| Name | Sequence 5’-3’ | Label |
| --- | --- | --- |
| Mut1 | GGGCTGGGGGGAGGGTGGCCGCGGTGGAGCCACGGGGCGGGCTTGGCTTGGTGTGACGGCGGCTGCGGCGGCGG | 5' Biotin |
| Mut2 | CGGCTGCGGCGACGGTGGCCGCGGTGGAGCCACGGGGCGGGCTTGGCTTGGTGTGACGGGGGCTGGGGGGGGGG | 5' Biotin |
| Mut1+2 | GGGCTGGGGGGAGGGTGGCCGCGGTGGAGCCACGGGGCGGGCTTGGCTTGGTGTGACGGGGGCTGGGGGGGGGG | 5' Biotin |

**Supplementary Figures**

**
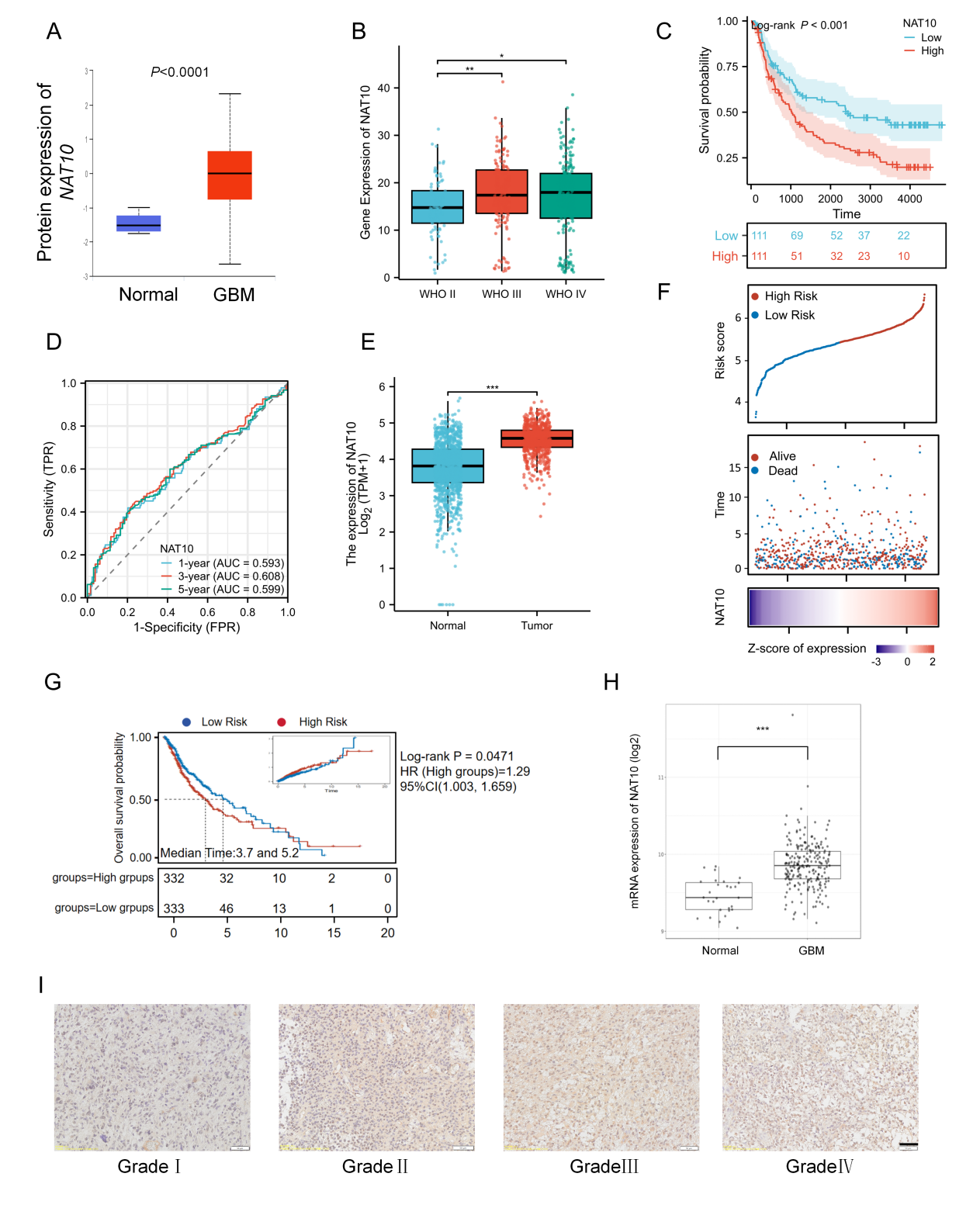
**

**Figure S1.** A. The CPTAC database was used to analyze the protein expression levels of NAT10 in GBM. B. The CGGA database was employed to assess the expression of NAT10 in tumors. C. Kaplan-Meier survival analysis was performed on the CGGA dataset, and the log-rank test was applied for comparison between different groups. D. The ROC curve and AUC for NAT10 were plotted, with a higher AUC value indicating better predictive power. E. The TCGA database was employed to assess the expression of NAT10 in glioma. F. Risk score, survival time, and survival status of NAT10 in the TCGA dataset. The scatter plot at the top displays the risk scores, ordered from low to high, with different colors representing distinct groups. The scatter plot distribution reflects the correlation between risk score, survival time, and survival status for different samples. The heatmap at the bottom shows the expression levels of NAT10. G. Kaplan-Meier survival analysis was conducted for different groups in the TCGA dataset. H. The Rembrandt database was employed to assess the expression of NAT10 in tumors. I. Representative immunohistochemical images of NAT10 protein expression in different grade glioma patients. ****p*<0.001, ***p*<0.01, **p*<0.05. (Student’s t test).


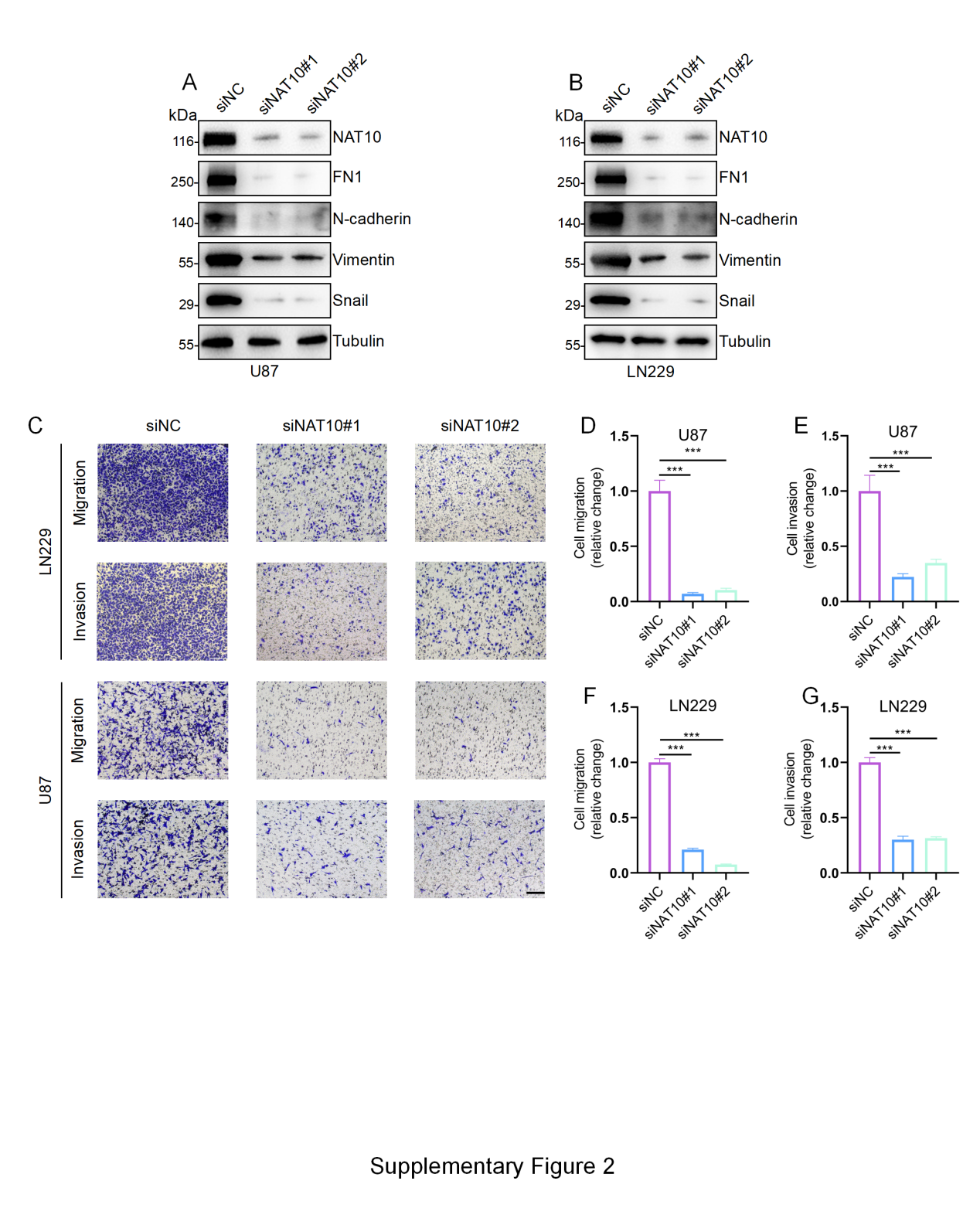


**Figure S2.** A and B. Western blot analysis of FN1, N-cadherin, Vimentin, and Snail expression in U87 and LN229 cells after siNAT10. C-G. Transell assay was used to detect the migration and invasion ability of tumor cells after small interference knockdown of NAT10. Error bars represent the mean ± SD. n = 3. ****p*<0.001. (Student’s t test).


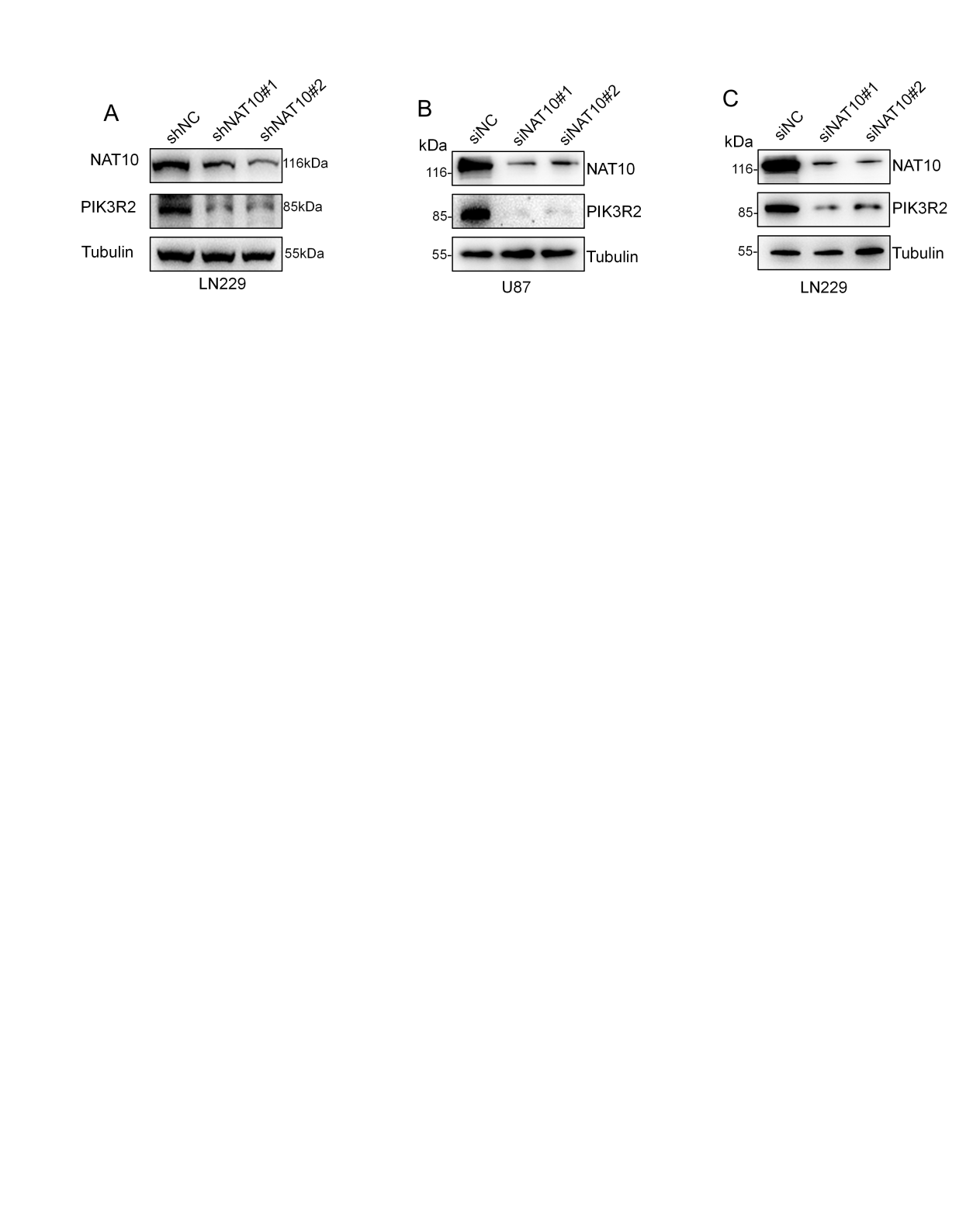


**Figure S3.** A-C. Changes in PIK3R2 expression levels assessed by Western blot following NAT10 knockdown.


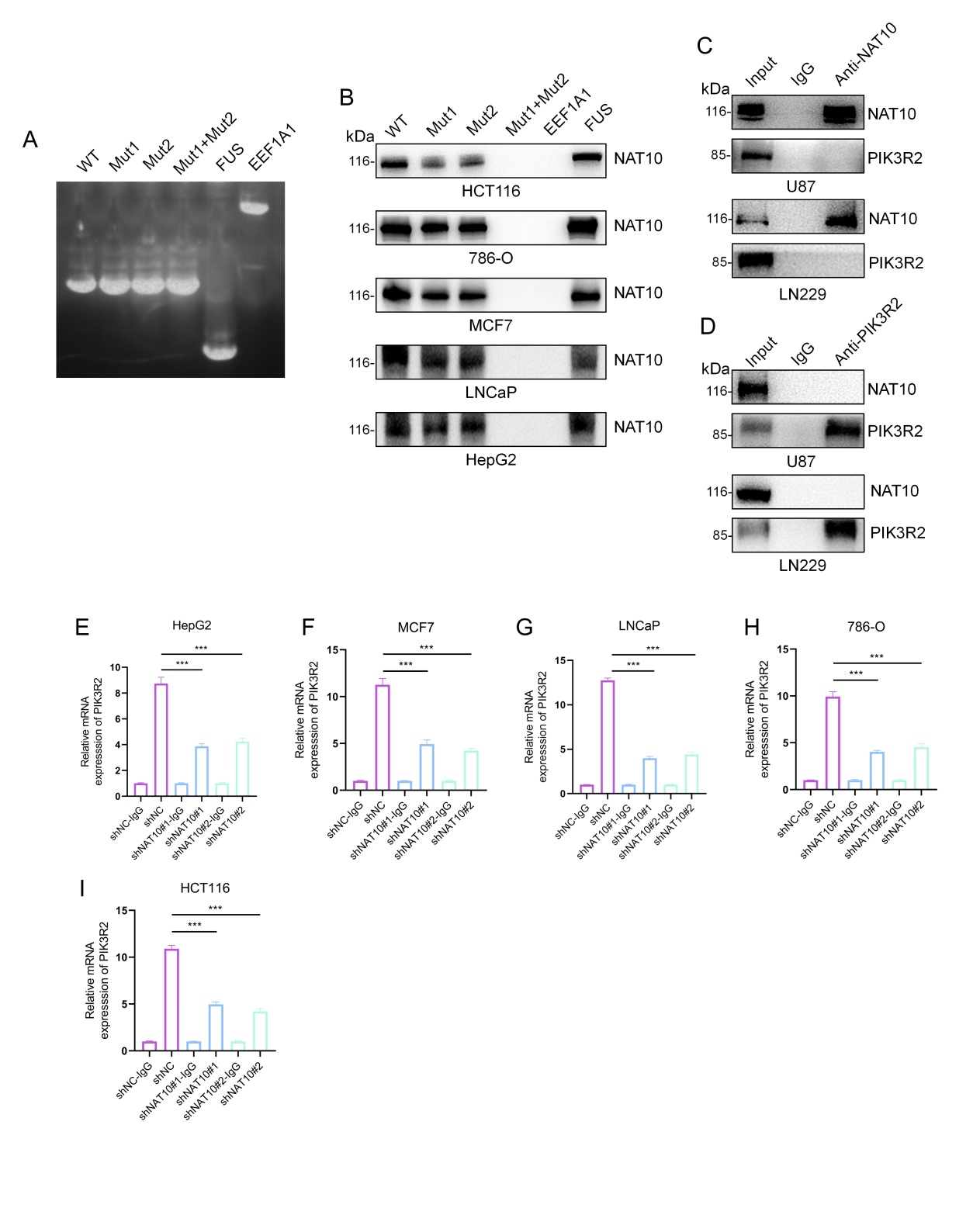


**Figure S4.**  A. Probe for detection of PIK3R2, FUS and EEF1A1 plasmid synthesis by gel electrophoresis. B. RNA pull-down assays were performed to verify specific modification site binding in HCT116, 786-O, MCF7, LNCaP, and HepG2 cells. C. NAT10 immunoprecipitation (IP) in U87 and LN229 cells followed by Western blot detection of NAT10 and PIK3R2. D. PIK3R2 immunoprecipitation (IP) in U87 and LN229 cells followed by Western blot detection of NAT10 and PIK3R2. E-I. ac4C-RIP assay detecting mRNA levels of ac4C-modified PIK3R2 in HCT116, 786-O, MCF7, LNCaP, and HepG2 cells after NAT10 knockdown. Error bars represent the mean ± SD. n = 3. ****p*<0.001. (Student’s t test).


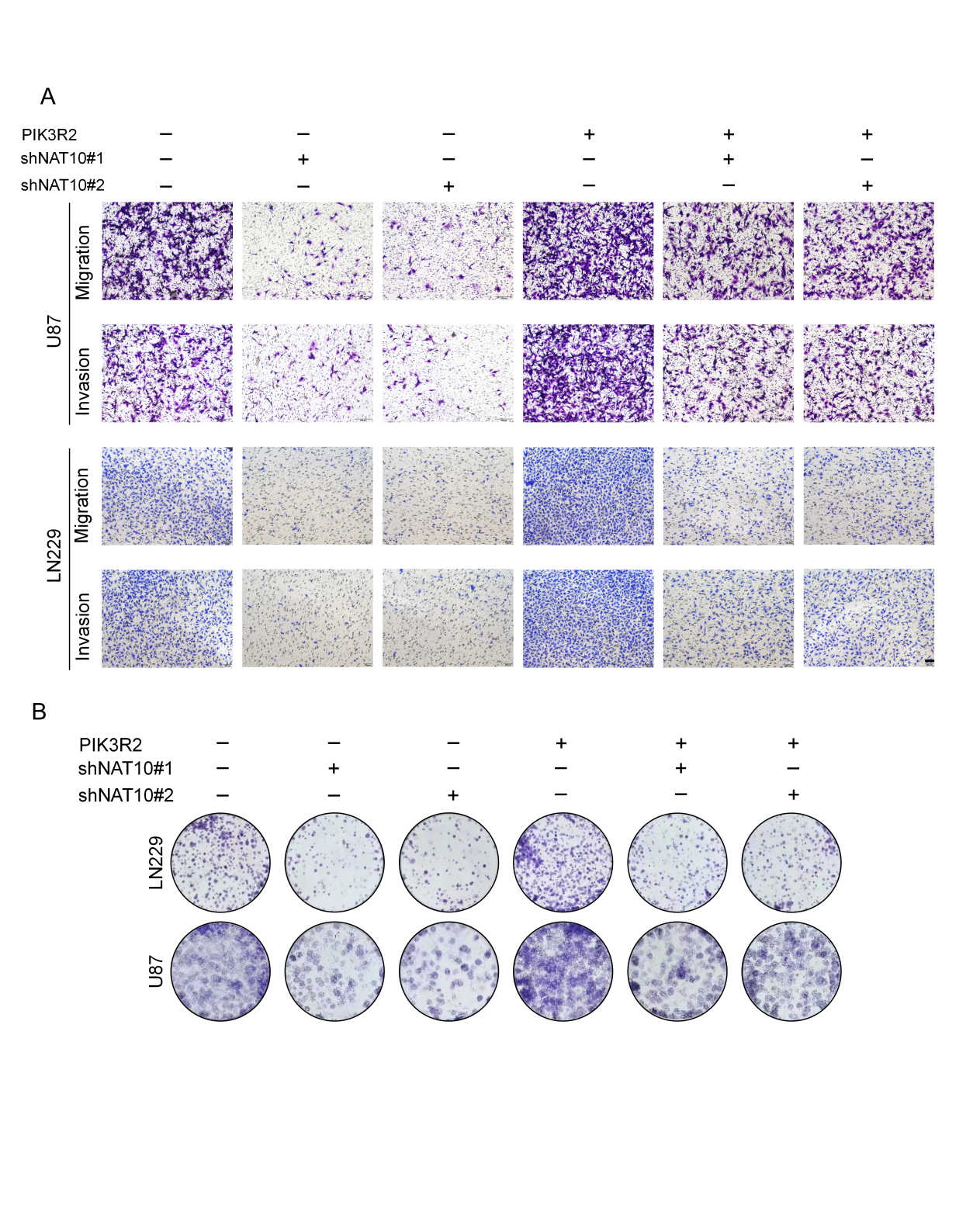


**Figure S5.** A. Transwell assays evaluating migration and invasion abilities of U87 and LN229 cells with various treatments: shNC, shNAT10, shNAT10 + PIK3R2, and PIK3R2. B. CCK-8 and colony formation assays assessing cell proliferation in U87 and LN229 cells under the same treatment conditions.


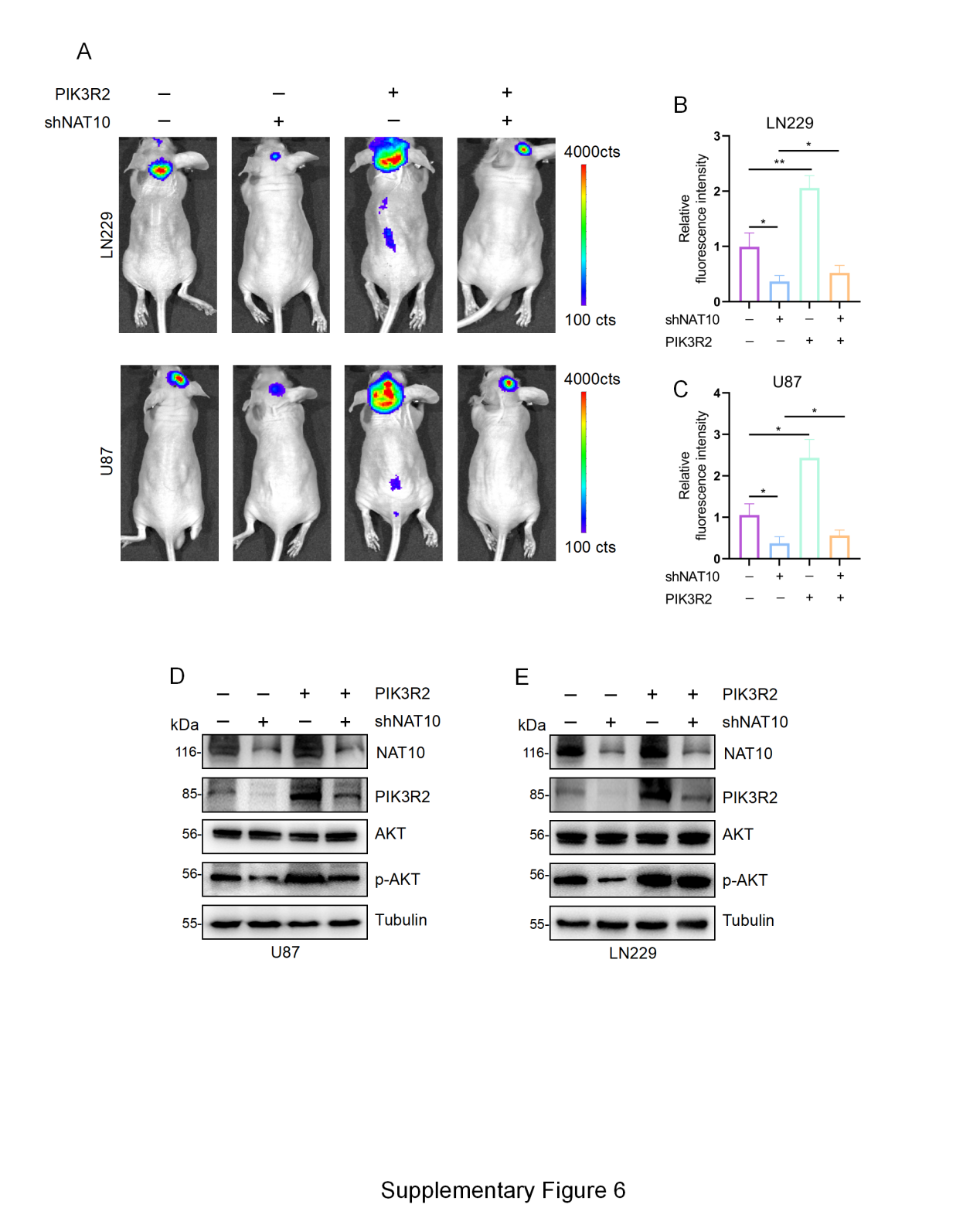


**Figure S6.** A-C. LN229 and U87 cells with shNC, shNAT10, PIK3R2 and shNAT10+PIK3R2 were orthotopically transplanted into the right striatum of mice (n=5 per group). Tumor growth was evaluated by measuring fluorescence intensity using an in vivo imaging system. D and E. Western blotting was performed to detect the protein levels of NAT10, PIK3R2, AKT, and P-AKT. Error bars represent the mean ± SD. n = 5. ***p*<0.01, **p*<0.05. (Student’s t test).


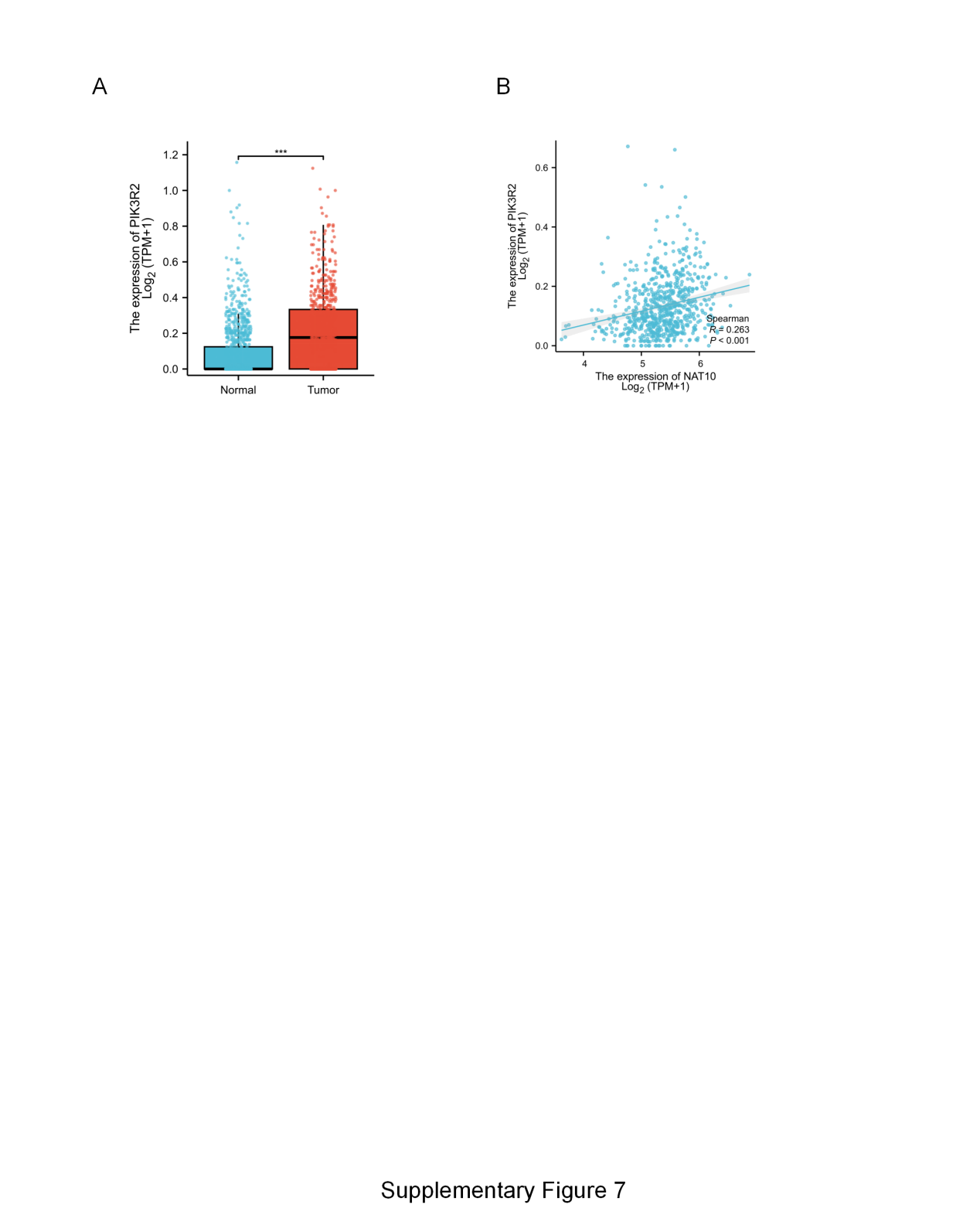


**Figure S7. A**. The TCGA database was employed to assess the expression of PIK3R2 in tumors. B. The mRNA expression levels of NAT10 and PIK3R2 in the glioma dataset were subjected to linear correlation analysis.
