# Supplementary material for: NAT10-mediated N4-acetylcytidine (ac4C) modification of PIK3R2 mRNA promotes malignant progression of glioblastoma
Source: Cell Death Dis. 2025 Dec 17;17(1):106. doi: 10.1038/s41419-025-08328-y (PMC12848064; doi:10.1038/s41419-025-08328-y)
Supplement: Supplementary file 2 — Orignal WB [file 41419_2025_8328_MOESM2_ESM.pdf]

C

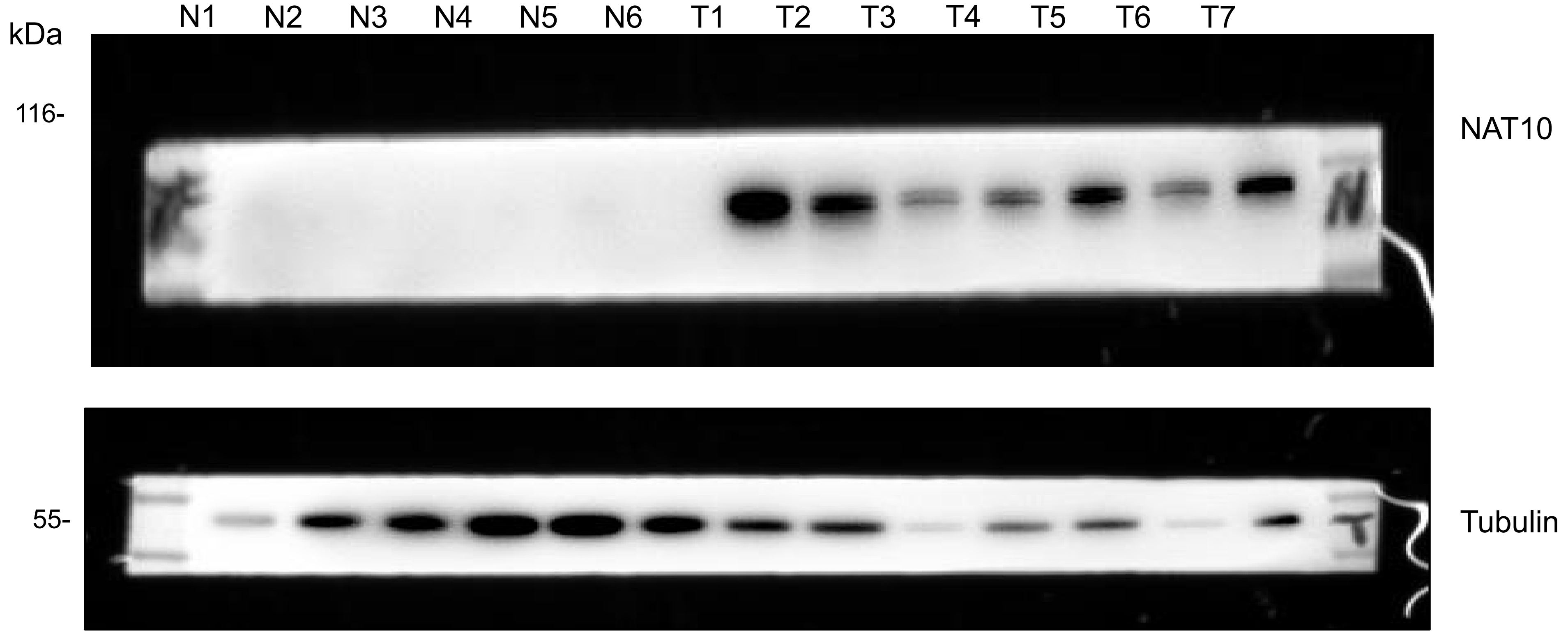

Figure 1

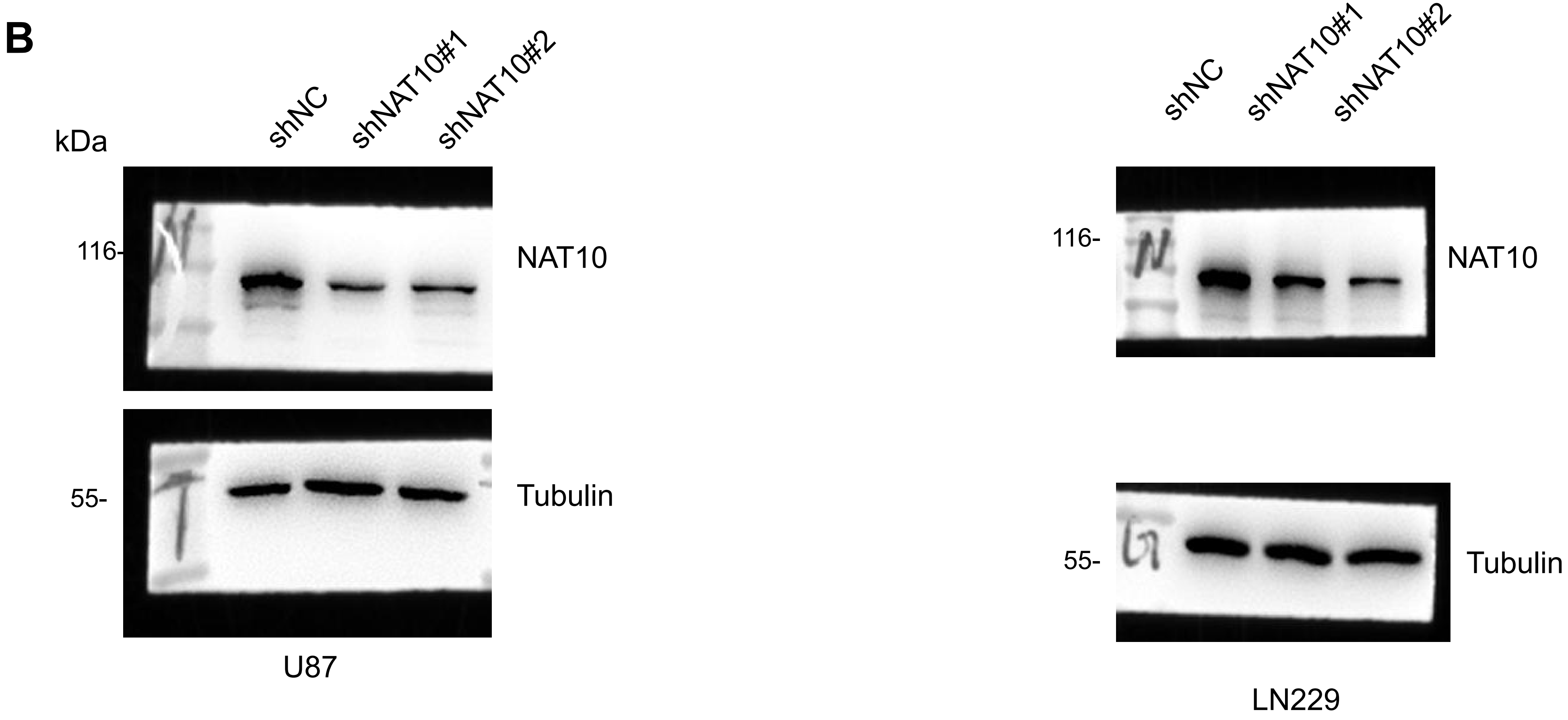
B
kDa
shNC
shNAT10#1
shNAT10#2
116-
NAT10
55-
Tubulin
U87
shNC
shNAT10#1
shNAT10#2
116-
NAT10
55-
Tubulin
LN229

Figure 2

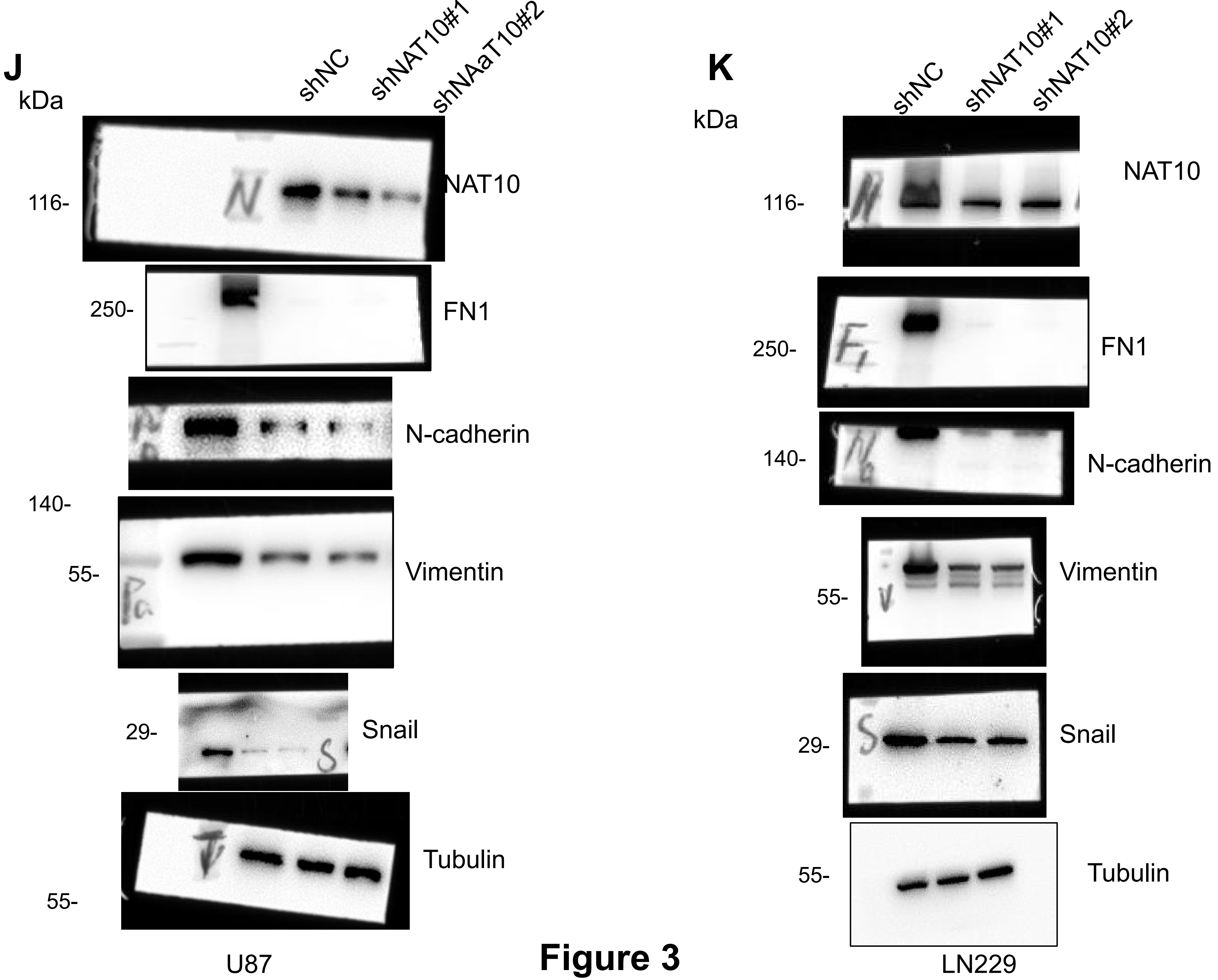
J
kDa
shNC
shNAT10#1
shNAaT10#2
116-
NAT10
250-
FN1
N-cadherin
140-
55-
Vimentin
29-
Snail
Tubulin
55-
U87
K
kDa
shNC
shNAT10#1
shNAT10#2
116-
NAT10
250-
FN1
140-
N-cadherin
55-
Vimentin
29-
Snail
55-
Tubulin
LN229

Figure 3

L

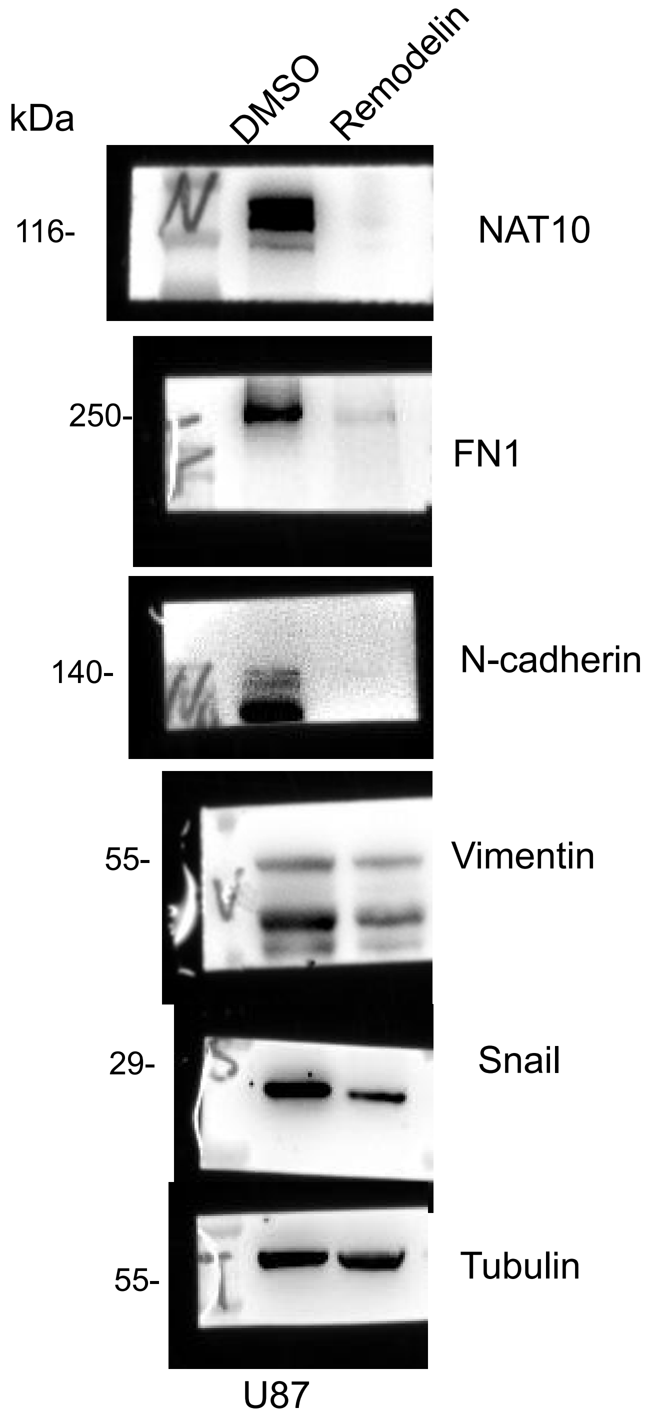
kDa
DMSO
Remodelin
116-
NAT10
250-
FN1
140-
N-cadherin
55-
Vimentin
29-
Snail
55-
Tubulin
U87

M

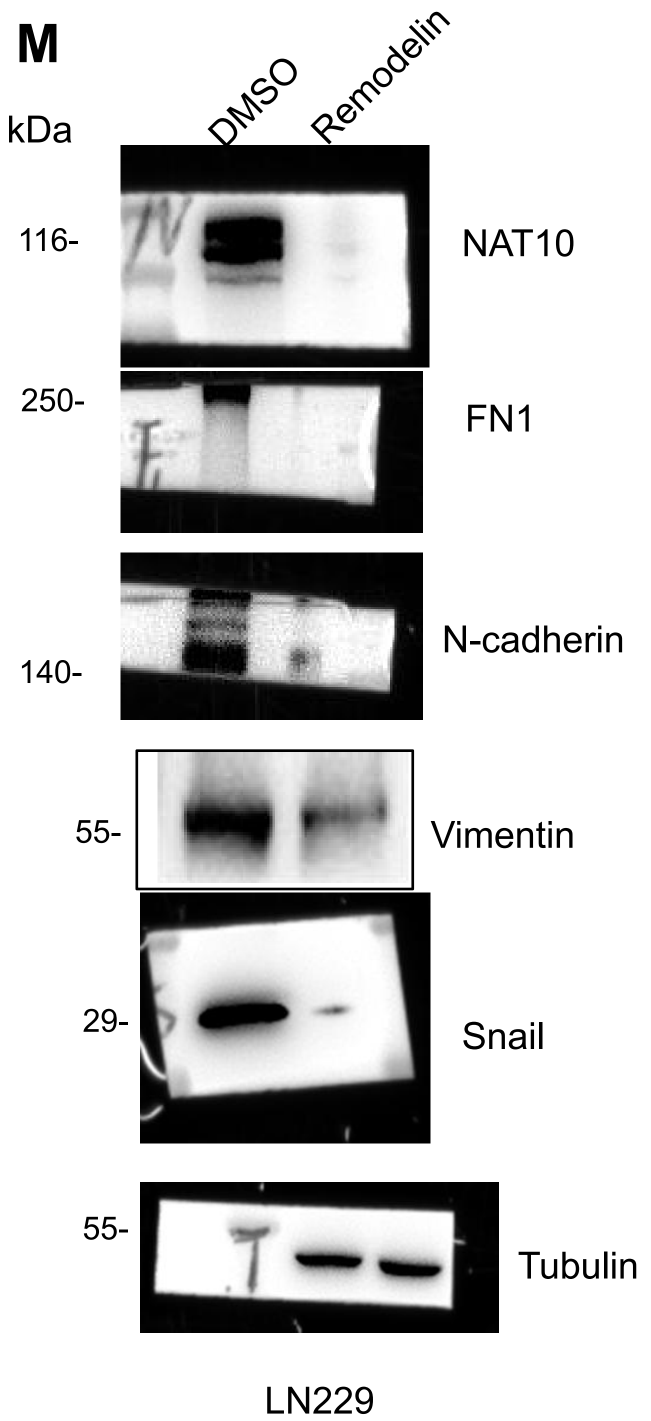
kDa
DMSO
Remodelin
116-
NAT10
250-
FN1
140-
N-cadherin
55-
Vimentin
29-
Snail
55-
Tubulin
LN229

Figure 3

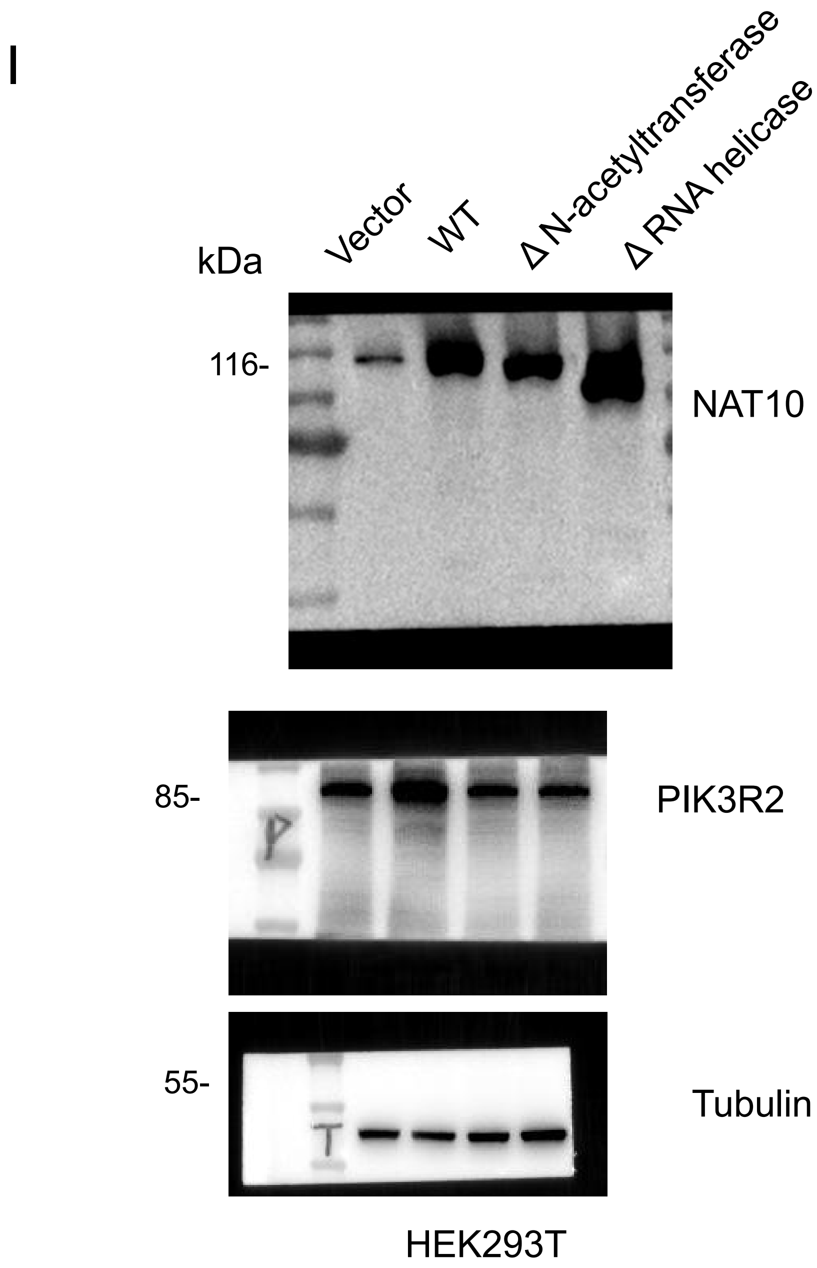
I
Vector
WT
Δ N-acetyltransferase
Δ RNA helicase
kDa
116-
NAT10
85-
PIK3R2
55-
Tubulin
HEK293T

Figure 4

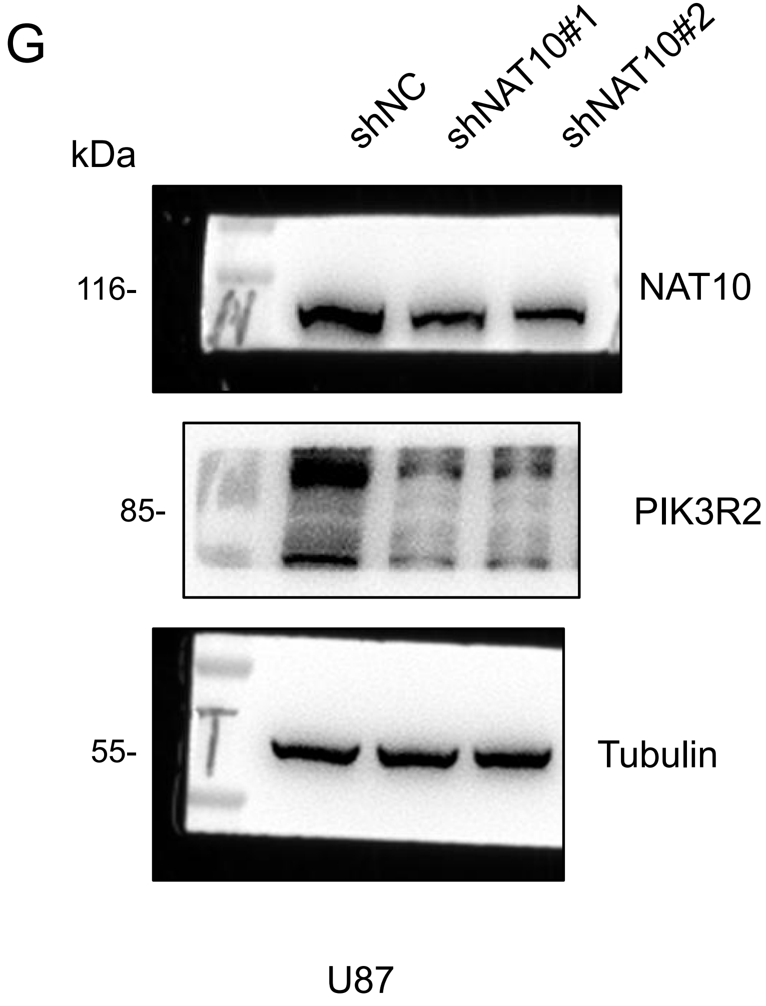

G
shNC
shNAT10#1
shNAT10#2
kDa
116-
NAT10
85-
PIK3R2
55-
Tubulin
U87

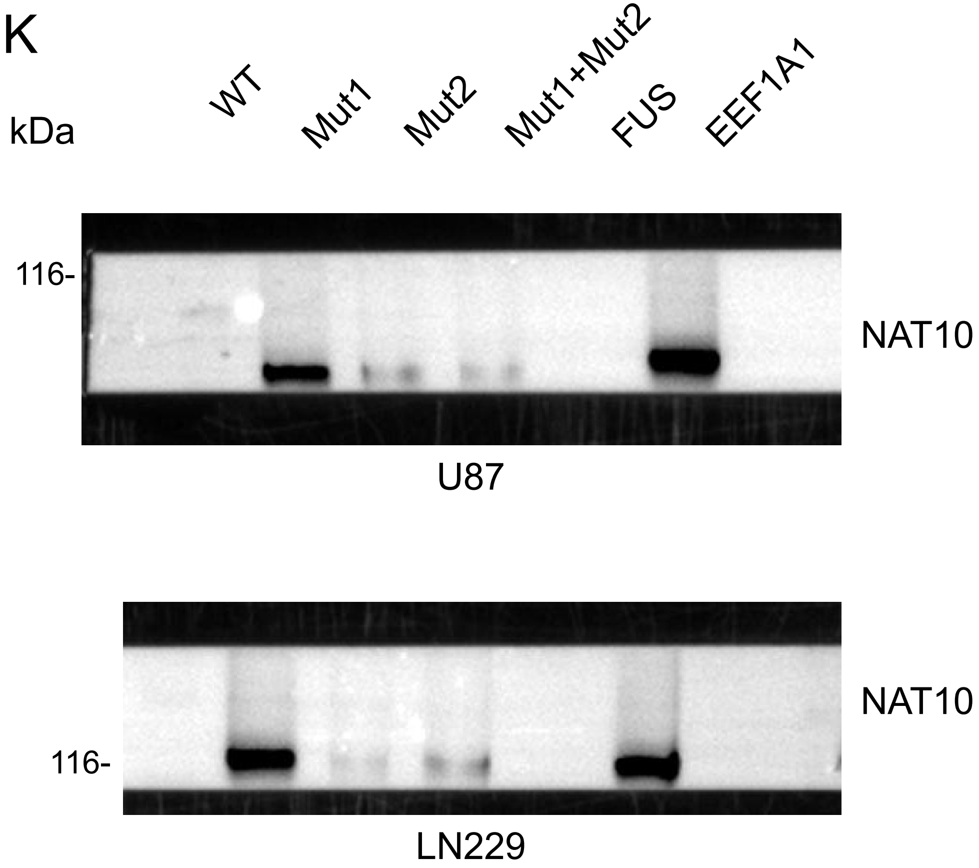

K
WT
Mut1
Mut2
Mut1+Mut2
FUS
EEF1A1
kDa
116-
NAT10
U87
NAT10
116-
LN229

Figure 4

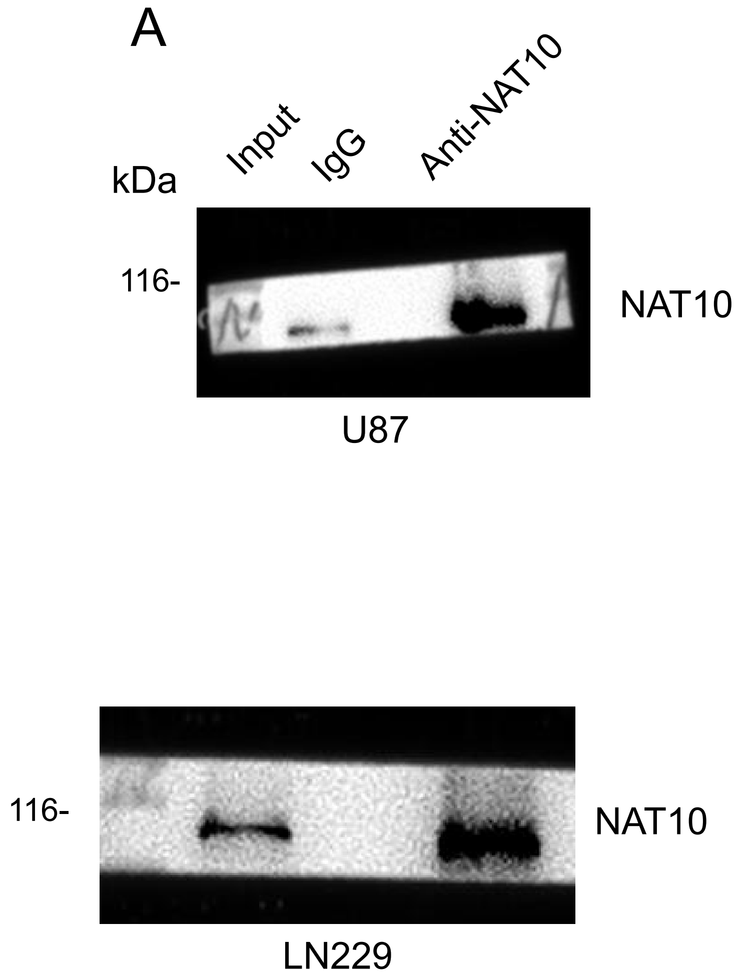

Figure 5

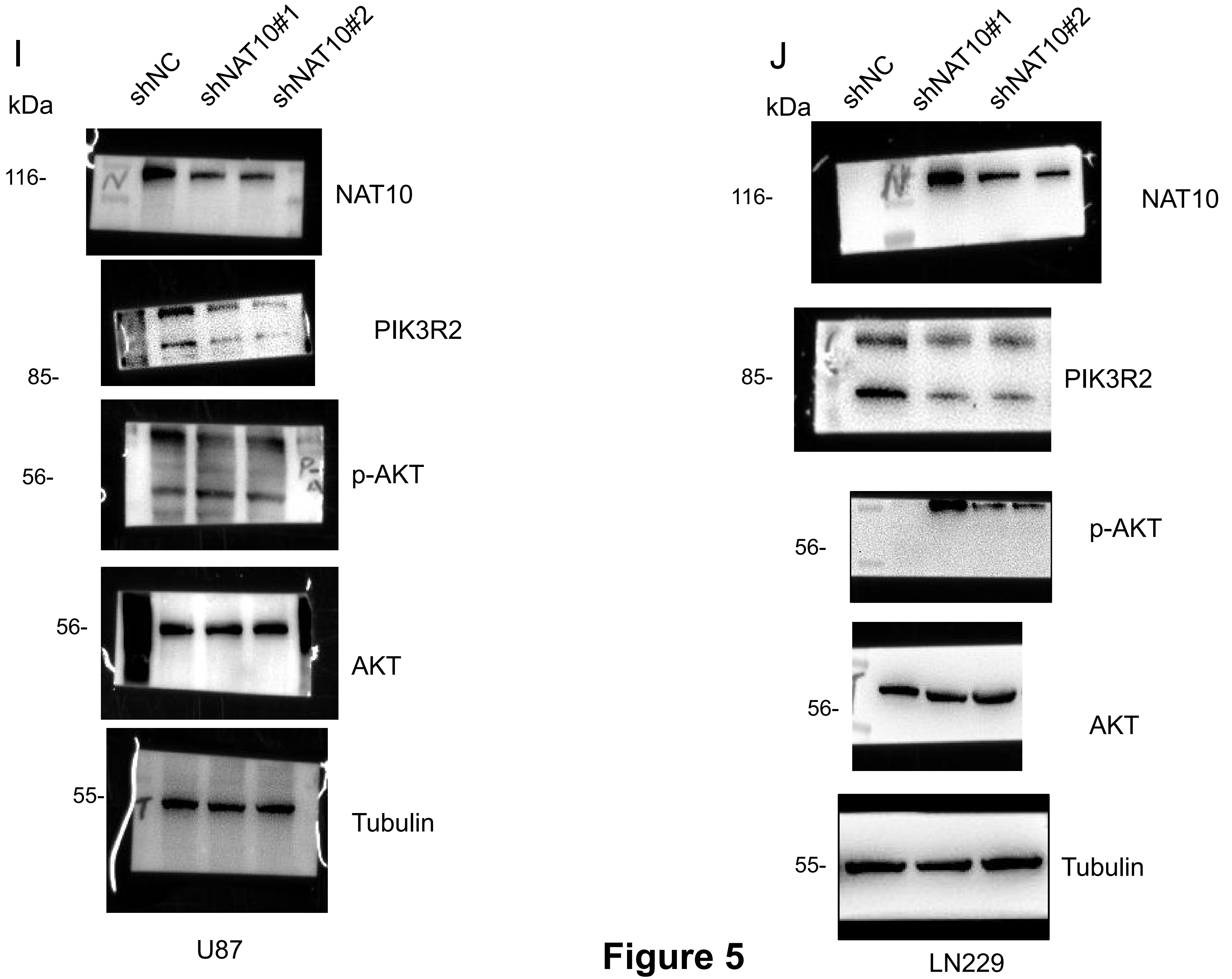
I
kDa
shNC
shNAT10#1
shNAT10#2
116-
NAT10
PIK3R2
85-
56-
p-AKT
56-
AKT
55-
Tubulin
U87
J
kDa
shNC
shNAT10#1
shNAT10#2
116-
NAT10
85-
PIK3R2
56-
p-AKT
56-
AKT
55-
Tubulin
LN229

Figure 5

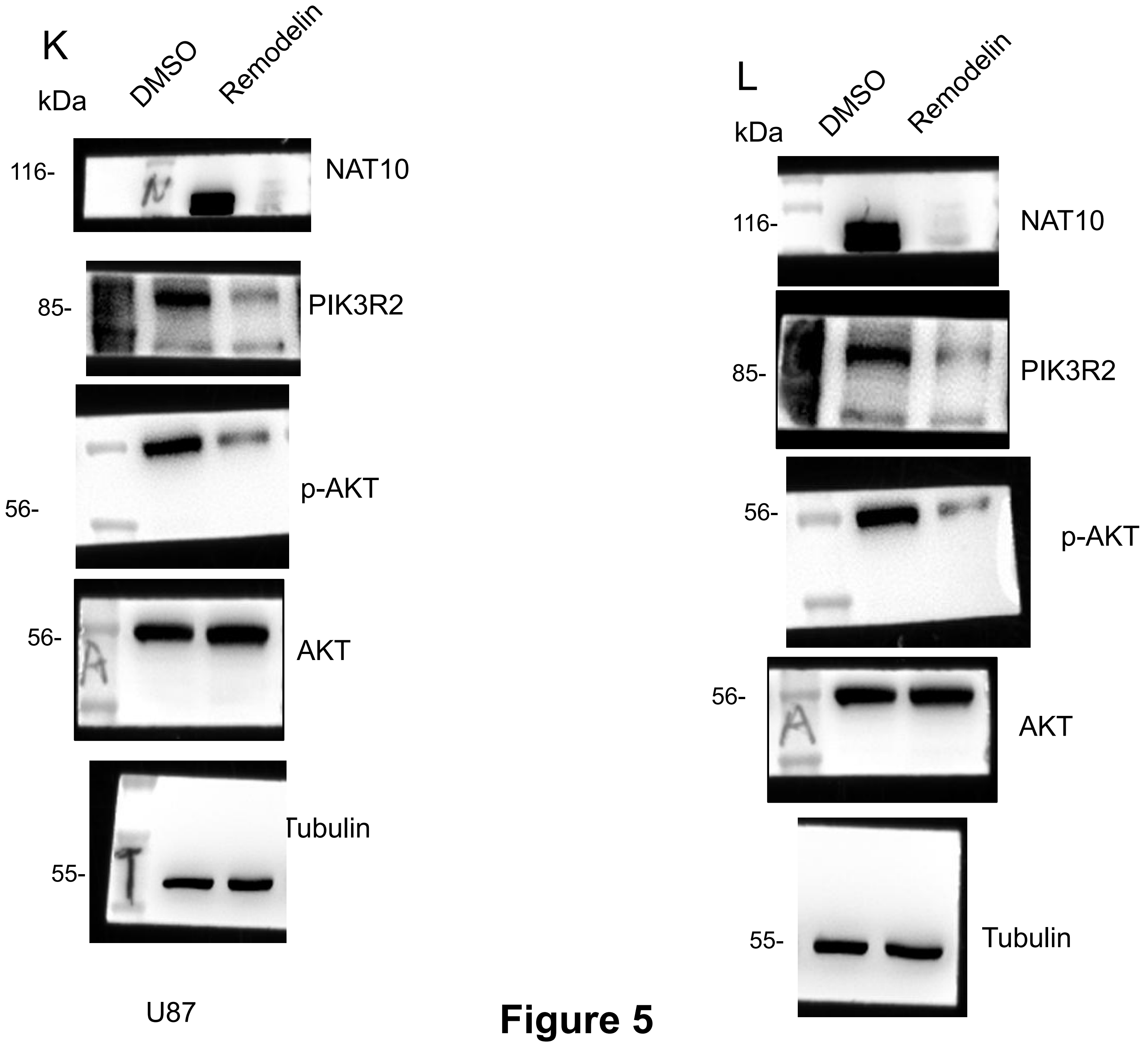
K
kDa
DMSO
Remodelin
116-
NAT10
85-
PIK3R2
56-
p-AKT
56-
AKT
55-
Tubulin
U87
L
kDa
DMSO
Remodelin
116-
NAT10
85-
PIK3R2
56-
p-AKT
56-
AKT
55-
Tubulin

Figure 5

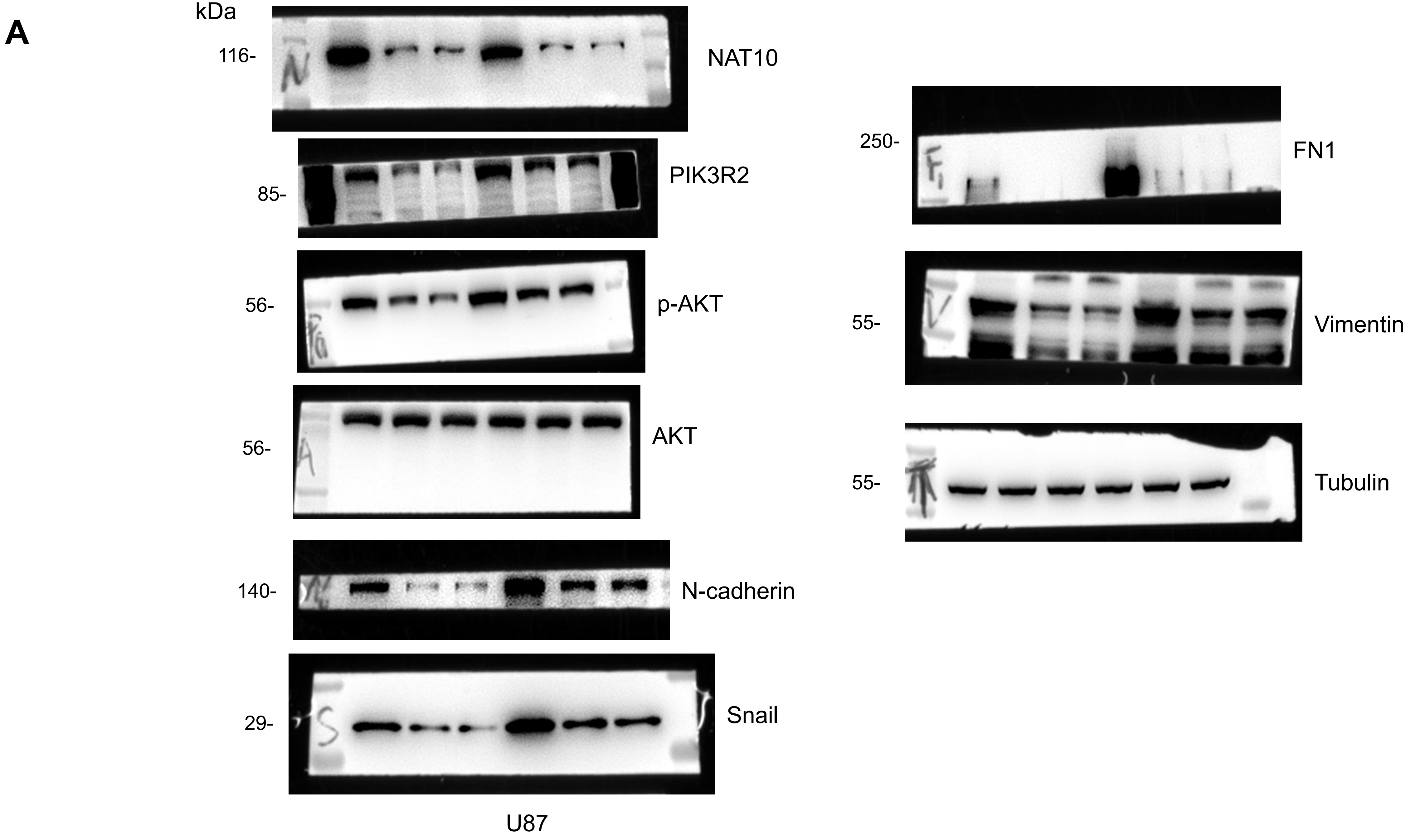
A
kDa
116-
NAT10
85-
PIK3R2
56-
p-AKT
56-
AKT
140-
N-cadherin
29-
Snail
U87
250-
FN1
55-
Vimentin
55-
Tubulin

Figure 6

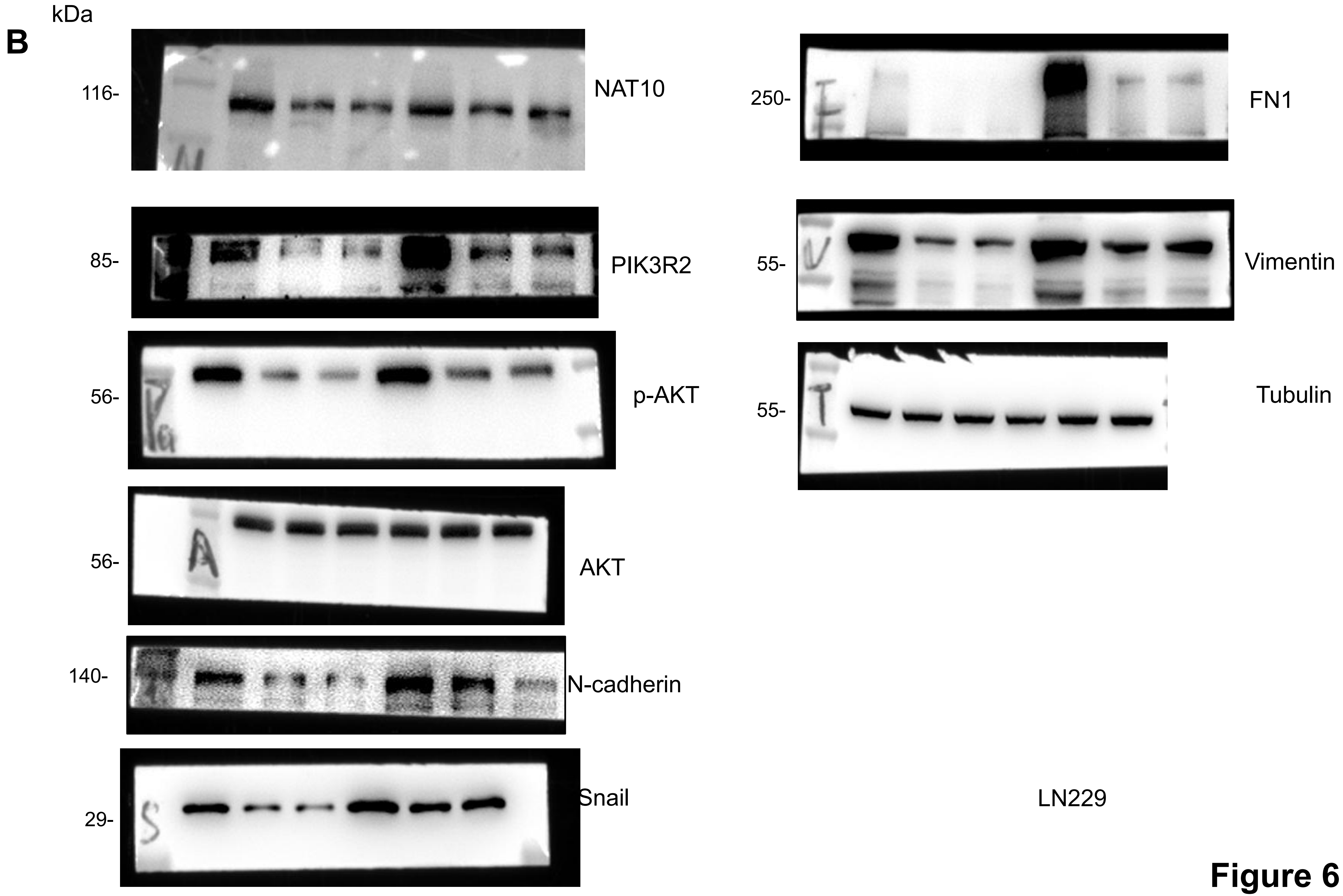
B
kDa
116-
NAT10
85-
PIK3R2
56-
p-AKT
56-
AKT
140-
N-cadherin
29-
Snail
250-
FN1
55-
Vimentin
55-
Tubulin
LN229

Figure 6

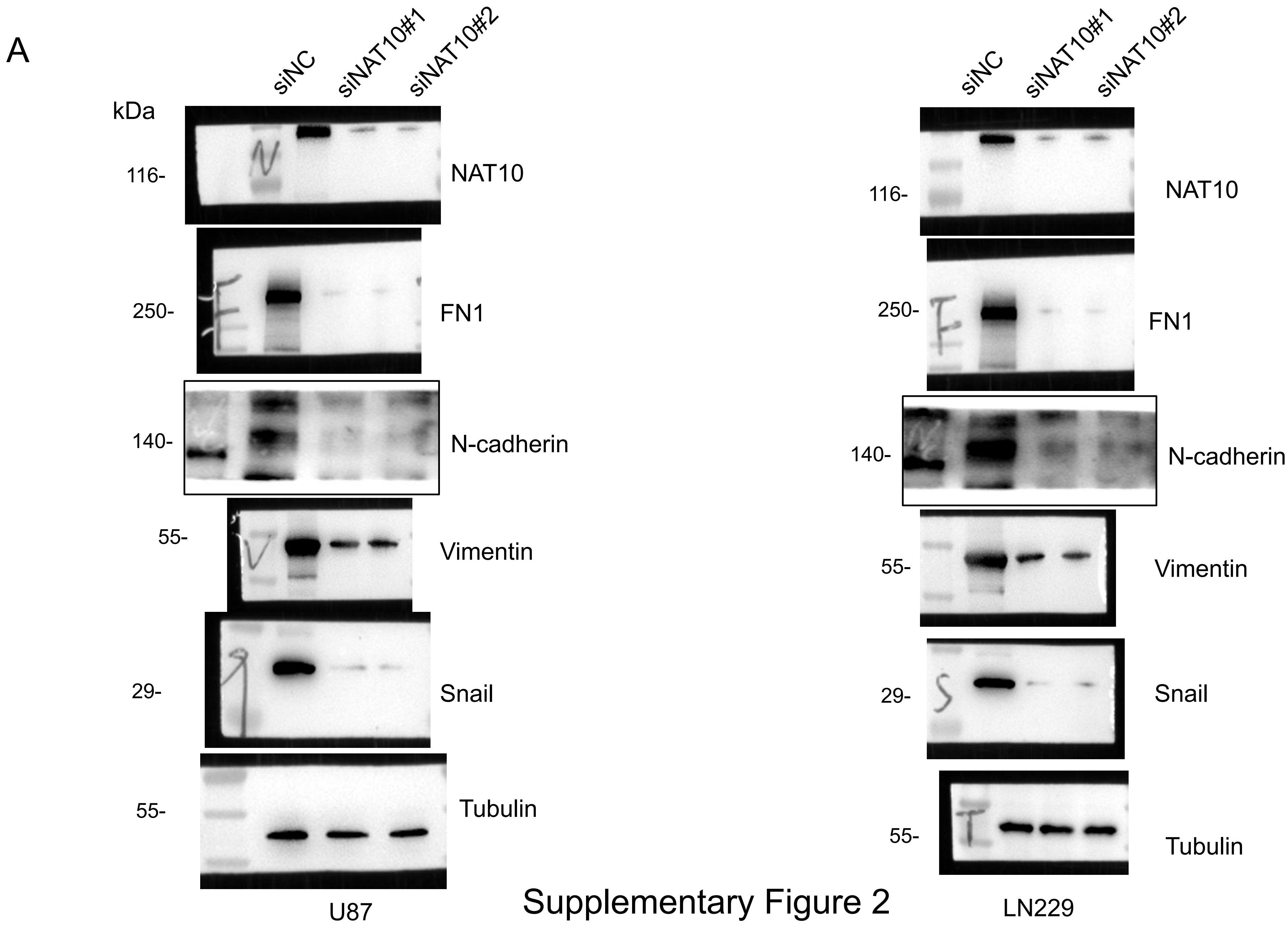

Supplementary Figure 2

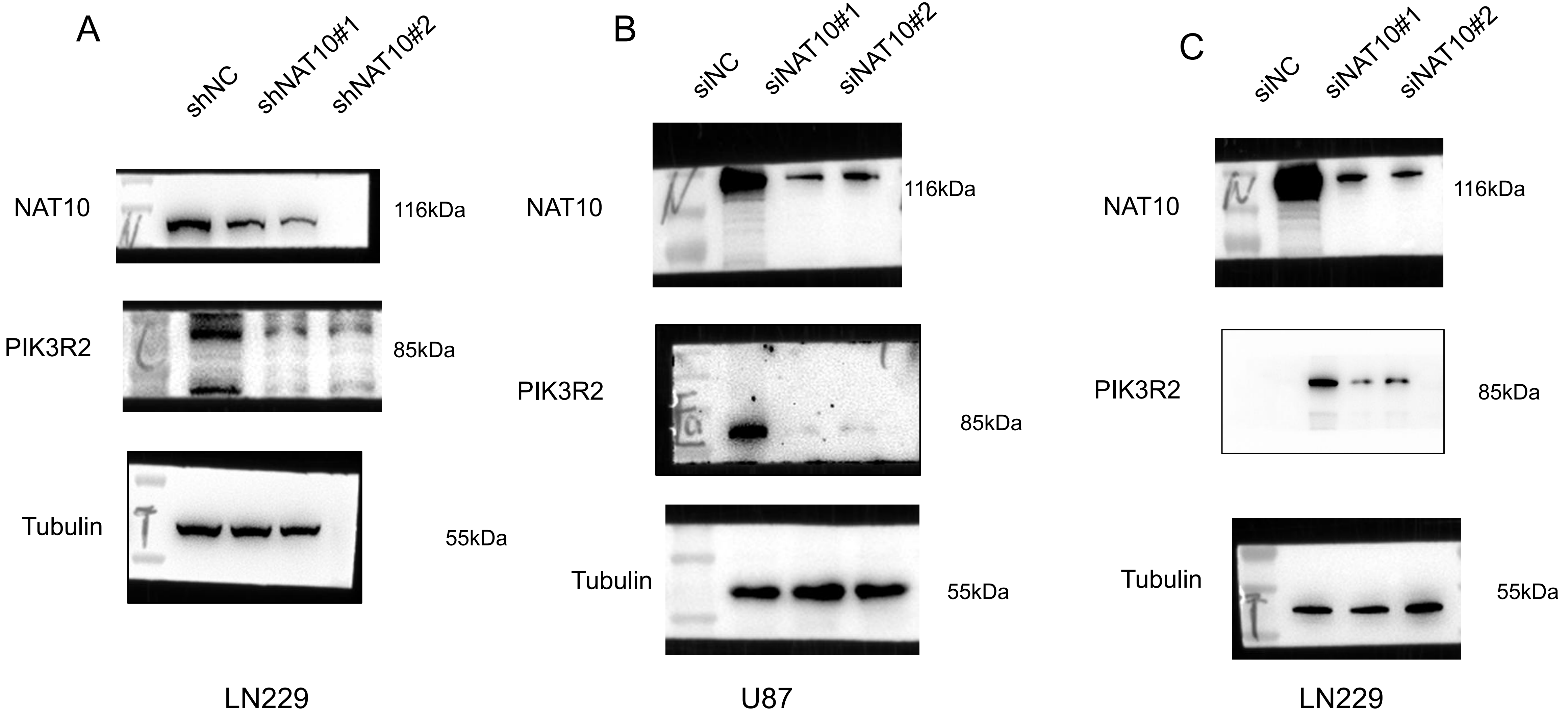

Supplementary Figure 3

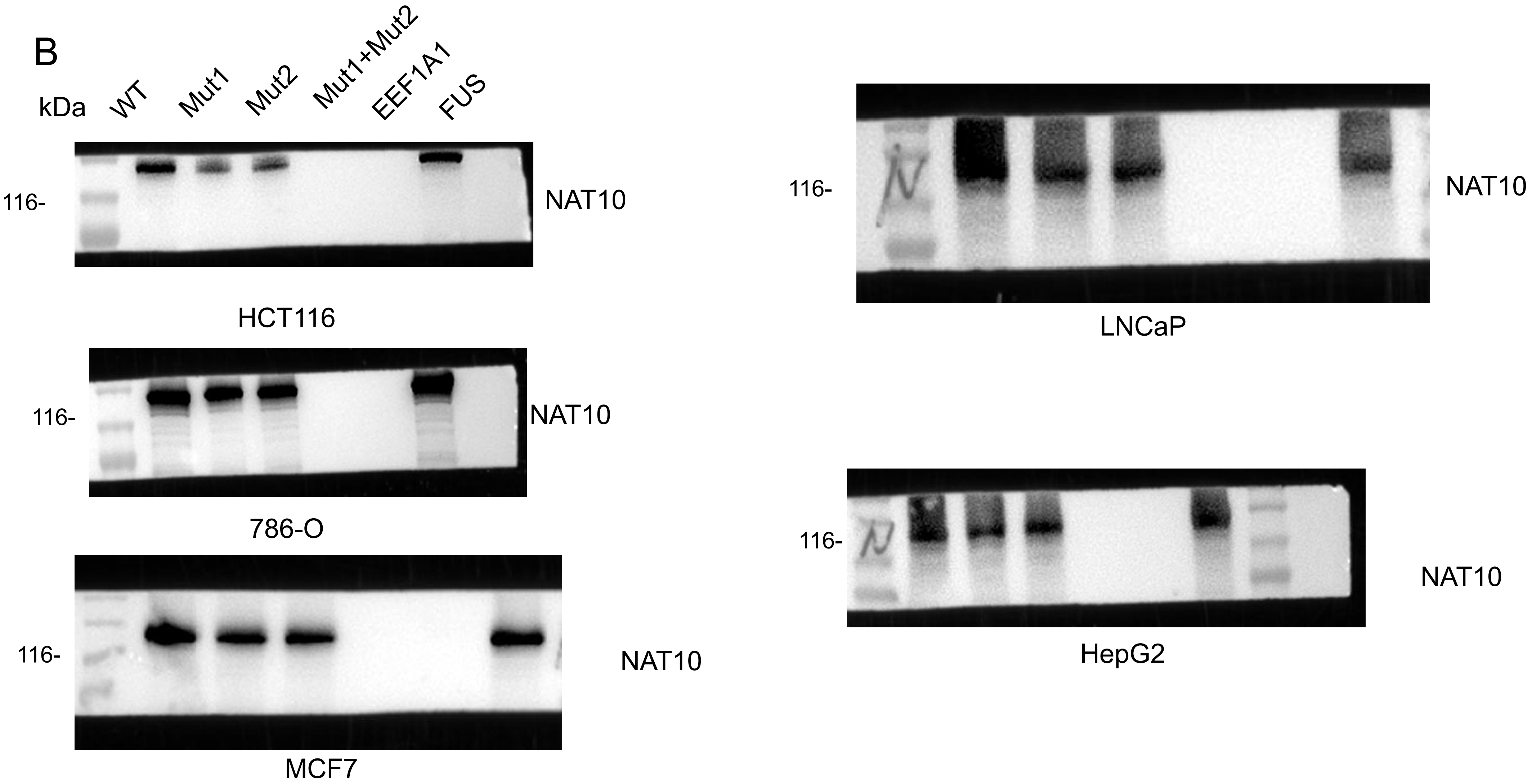

Supplementary Figure 4

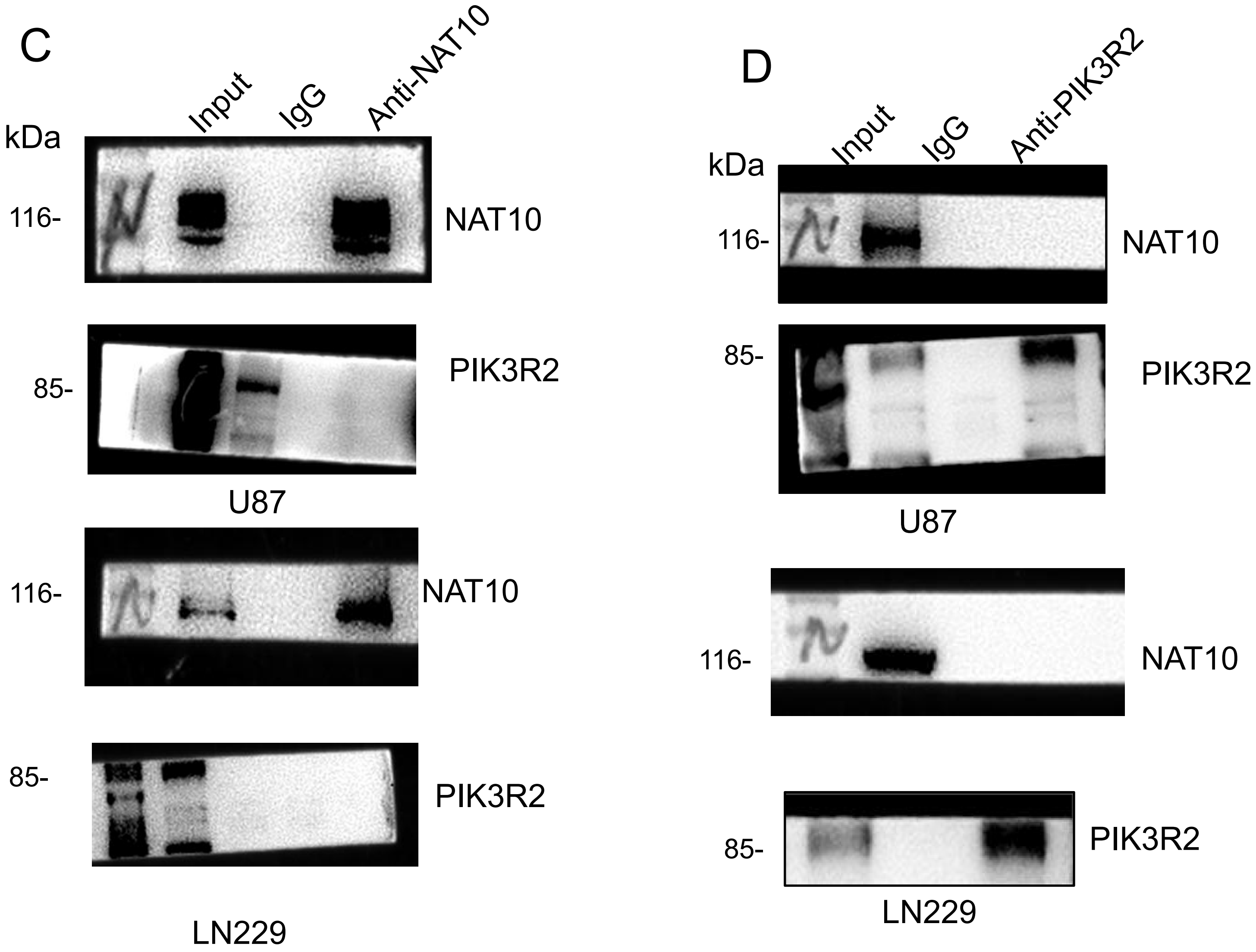

Supplementary Figure 4

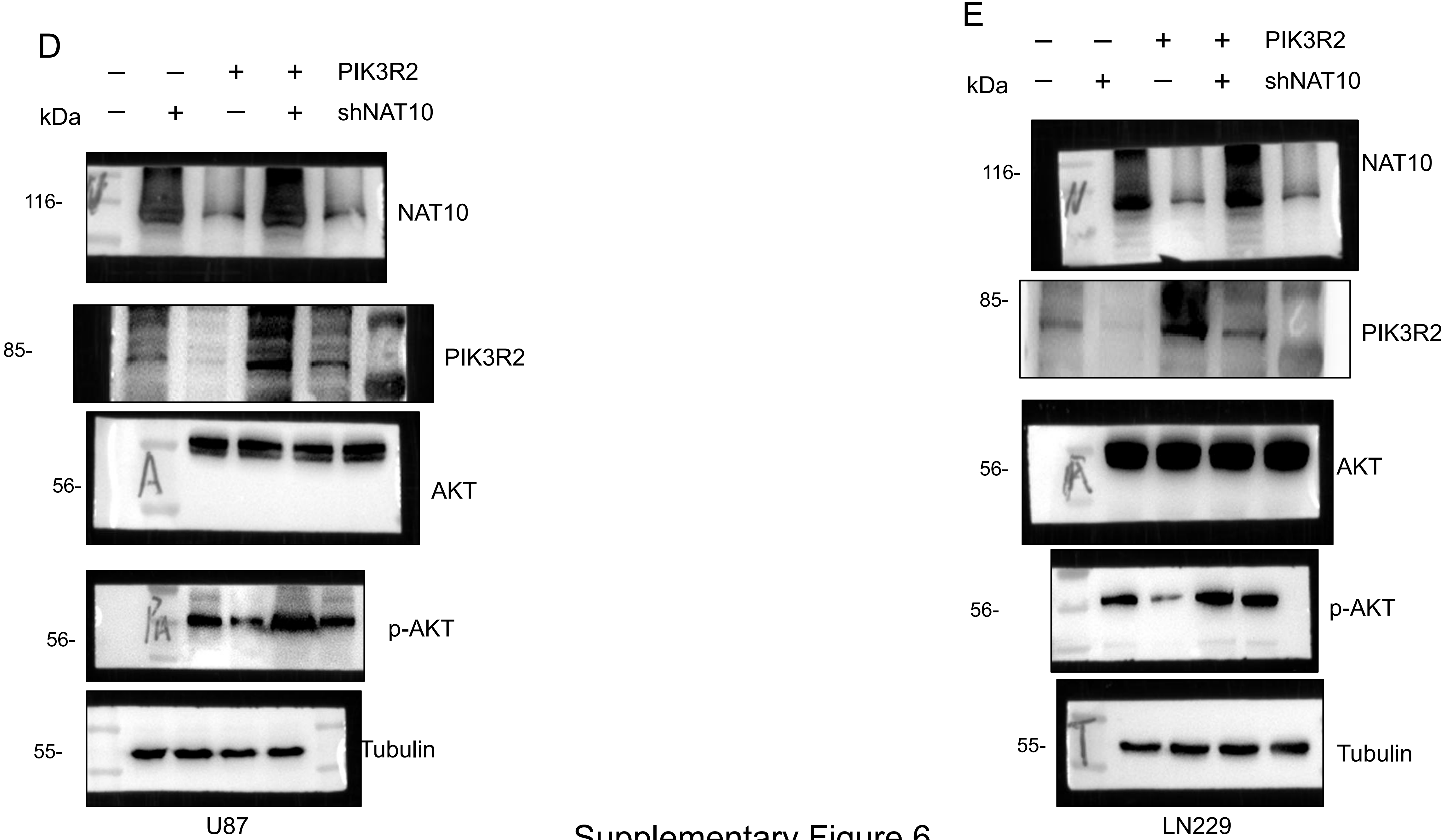

Supplementary Figure 6
